# Comparing antigenaemia- and microfilaraemia as criteria for stopping decisions in lymphatic filariasis elimination programmes in Africa

**DOI:** 10.1371/journal.pntd.0010953

**Published:** 2022-12-12

**Authors:** Wilma A. Stolk, Luc E. Coffeng, Fatorma K. Bolay, Obiora A. Eneanya, Peter U. Fischer, T. Déirdre Hollingsworth, Benjamin G. Koudou, Aboulaye Méité, Edwin Michael, Joaquin M. Prada, Rocio M. Caja Rivera, Swarnali Sharma, Panayiota Touloupou, Gary J. Weil, Sake J. de Vlas

**Affiliations:** 1 Department of Public Health, Erasmus MC, University Medical Center Rotterdam, Rotterdam, The Netherlands; 2 National Public Health Institute of Liberia (NPHIL), Monrovia, Liberia; 3 Infectious Diseases Division, Department of Medicine, Washington University School of Medicine, St. Louis, Missouri, United States of America; 4 Big Data Institute, Li Ka Shing Centre for Health Information and Discovery, University of Oxford, Oxford, United Kingdom; 5 Centre Suisse de Recherches Scientifiques en Côte d’Ivoire, Abidjan, Abidjan, Côte d’Ivoire; 6 Laboratoire de Cytologie et Biologie Animale, UFR Science de la Nature, Université Nangui Abrogoua Abidjan, Abidjan, Côte d’Ivoire; 7 Programme National de Lutte contre les Maladies Tropicales Négligées à Chimiothérapie Préventive, Abidjan, Côte d’Ivoire; 8 Center for Global Health Infectious Disease Research, University of South Florida, Tampa, Florida, United States of America; 9 Faculty of Health and Medical Sciences, University of Surrey, Guildford, United Kingdom; 10 Department of Biological Sciences, University of Notre Dame, South Bend, Indiana, United States of America; 11 Christian Medical College, IDA Scudder Rd, Vellore, Tamil Nadu, India; 12 Department of Statistics, University of Warwick, Coventry, United Kingdom; 13 School of Mathematics, University of Birmingham, Birmingham, United Kingdom; University Hospital Bonn: Universitatsklinikum Bonn, GERMANY

## Abstract

**Background:**

Mass drug administration (MDA) is the main strategy towards lymphatic filariasis (LF) elimination. Progress is monitored by assessing microfilaraemia (Mf) or circulating filarial antigenaemia (CFA) prevalence, the latter being more practical for field surveys. The current criterion for stopping MDA requires <2% CFA prevalence in 6- to 7-year olds, but this criterion is not evidence-based. We used mathematical modelling to investigate the validity of different thresholds regarding testing method and age group for African MDA programmes using ivermectin plus albendazole.

**Methodolgy/Principal findings:**

We verified that our model captures observed patterns in Mf and CFA prevalence during annual MDA, assuming that CFA tests are positive if at least one adult worm is present. We then assessed how well elimination can be predicted from CFA prevalence in 6-7-year-old children or from Mf or CFA prevalence in the 5+ or 15+ population, and determined safe (>95% positive predictive value) thresholds for stopping MDA. The model captured trends in Mf and CFA prevalences reasonably well. Elimination cannot be predicted with sufficient certainty from CFA prevalence in 6-7-year olds. Resurgence may still occur if all children are antigen-negative, irrespective of the number tested. Mf-based criteria also show unfavourable results (PPV <95% or unpractically low threshold). CFA prevalences in the 5+ or 15+ population are the best predictors, and post-MDA threshold values for stopping MDA can be as high as 10% for 15+. These thresholds are robust for various alternative assumptions regarding baseline endemicity, biological parameters and sampling strategies.

**Conclusions/Significance:**

For African areas with moderate to high pre-treatment Mf prevalence that have had 6 or more rounds of annual ivermectin/albendazole MDA with adequate coverage, we recommend to adopt a CFA threshold prevalence of 10% in adults (15+) for stopping MDA. This could be combined with Mf testing of CFA positives to ensure absence of a significant Mf reservoir for transmission.

## Author summary

Mass drug administration (MDA) of antifilarial drugs is the main strategy towards the elimination of lymphatic filariasis (LF), but defining when MDA can safely be stopped is challenging. Current stopping guidelines require that the prevalence of microfilaraemia (Mf) in the population falls below 1% or the prevalence of circulating filarial antigenaemia (CFA) in 6-and-7-year-old children falls below 2%. The evidence base underlying this threshold is limited. The accuracy of this threshold is hard to assess in field studies due to the long timespan between stopping MDA and the occurrence of either elimination or resurgence. We used mathematical modelling to assess how well elimination can be predicted at community-level from the Mf or CFA prevalence observed 1-year after the last MDA round and we determined safe stopping thresholds (>95% certainty that elimination is achieved). We found that the currently used CFA prevalence in 6–7 year-old children is a poor indicator for stopping decisions in LF elimination programmes implementing MDA with ivermectin plus albendazole, and that CFA prevalence among people aged 5 or 15 years and older is a much more reliable and practical indicator of elimination outcomes. Lymphatic filariasis elimination programmes should reconsider guidelines for stopping MDA.

## Introduction

Lymphatic filariasis (LF) is a mosquito-transmitted parasitic worm infection that prevails in tropical and subtropical regions across the world and is an important preventable cause of morbidity and disability [1]. Most LF is caused by the filarial nematode *Wuchereria bancrofti*. The long-lived adult worms reside in the human lymph system and are the main cause of morbidity (lymphoedema and hydrocele). Fertilized female worms release large numbers of microfilariae (worm’s offspring) into the bloodstream, where they can be picked up by mosquitoes to be passed on to other humans. Annual mass drug administration (MDA) is the mainstay of control, sometimes complemented by vector control measures such as the use of insecticide-treated bed nets.

The Global Programme to Eliminate Lymphatic Filariasis (GPELF) was initiated in 2000. It aims to eliminate LF as a public health problem by interrupting transmission through MDA of anti-filarial drugs (albendazole with ivermectin and/or diethylcarbamazine, with the three-drug combination restricted to specific settings warranting acceleration[2]) and by alleviating the suffering of people with clinical manifestations through morbidity management and disability prevention [3,4]. The rapid scale-up of MDA has led to a massive decline in the prevalence of LF infection. By 2020, 17 previously endemic countries no longer require MDA after reaching prevalence targets below which transmission is thought to be unsustainable [1]. Eight more have stopped all MDA and are now under surveillance to confirm elimination as a public health problem based on WHO criteria; 47 countries still require MDA in some or all of their endemic areas [5].

Progress towards elimination of *W*. *bancrofti* is monitored by examining trends in microfilaraemia (Mf) or circulating filarial antigenaemia (CFA) prevalence [6]. Based on experiences from China [7], a Mf prevalence below 1% is thought to lead to elimination where infection is transmitted by *Anopheles* or *Culex* mosquito species [6]. A lower threshold target is recommended where more efficient *Aedes* species transmit the parasites [6]. However, Mf prevalence surveys require microscopic examination of blood samples, and are extra cumbersome in areas such as Africa where night blood samples are needed because of nocturnal Mf periodicity. In contrast, CFA can be detected in day or night blood with a rapid point of care test that does not require microscopy [8,9]. The World Health Organization (WHO) therefore defined its stop-MDA recommendations based on CFA prevalence, which is to be assessed in 6-7-year-old children according to a specifically designed transmission assessment survey (TAS) [6]. MDA can be stopped if CFA prevalence is <2% where *Anopheles* or *Culex* are the main vectors and <1% where *Aedes* is the main vector [6]. Children are taken as sentinel group, as they should be free of LF infection if transmission is successfully interrupted. TAS is to be repeated two more times with intervals of two to three years between surveys, to validate elimination as a public health problem.

The TAS design was found to be practical, but whether this is sufficient to eliminate transmission and sustain the results in the long run remains uncertain [10]. There is concern that CFA prevalence in children is not a sensitive enough indicator for detecting residual transmission [11]. Children are not always exposed to the same number of infective mosquitoes as adults and may be CFA negative while transmission is ongoing in the adult population. Also, there is concern that the currently-used threshold is too stringent when the Alere Filariasis Test Strip (FTS) is used to detect CFA, because it is more sensitive than the previously used immunochromatographic card test (ICT); many FTS-positives are Mf negative and are therefore unlikely to contribute to transmission [12,13].

Mathematical models are useful tools to theoretically assess the validity of MDA stopping thresholds [14,15]. Modelling showed that the risk of LF resurgence is low in African settings where Mf prevalence in the 5+ population was reduced to <1% after five rounds of MDA with ivermectin plus albendazole [15]. Here, we use the established LYMFASIM model to assess the predictive value of Mf and CFA prevalence thresholds for the eventual occurrence of resurgence or elimination of LF, in relation to the sampled age group, for African LF elimination programmes implementing annual MDA with ivermectin plus albendazole. Before doing so, we verify that the model adequately captures the observed association between community-level Mf and CFA prevalence, before and during annual MDA.

## Methods

### The LYMFASIM model

LYMFASIM is an individual-based model, simulating LF transmission in a population (village or town) of hypothetical individuals [16,17]. The model tracks infection intensity (number of male and female adult worms; Mf density) for each individual, accounting for inter-individual variation in exposure to infection and participation in interventions. It captures the key processes involved in parasite biology, transmission and interventions. See section 1 in S1 Supplement for more information. LYMFASIM accounts for measurement variation in counting Mf, allowing for the occurrence of false-negative Mf counts for individuals with low worm burdens. For the current analysis, we assumed that all individuals with ≥1 mature male or female worm are CFA-positive, and that people turn CFA-negative within one month after a person’s last worm has died.

### Validation of model-predicted CFA prevalences

We validated model-predicted CFA prevalences using individual-level data from annually-treated communities from community intervention trials in Côte d’Ivoire (primary results will be published elsewhere) and Liberia[18] evaluating the effectiveness of annual versus biannual MDA on LF infection. Only for validating the model-predicted association between Mf and CFA prevalences at baseline (2013 for Côte d’Ivoire, 2014 for Liberia) we also used baseline data from biannually-treated communities.

We simulated the local history of control at community level, accounting for the timing of MDA rounds and surveys, and for the local bed net coverage since 2006. To obtain simulation runs across the spectrum of observed baseline endemicity levels, we performed a large number of runs per scenario, varying model parameters relating to setting-specific transmission conditions: the monthly biting rate, the degree of interindividual variation in exposure to mosquito bites, and external force-of-infection (i.e. the rate at which infection are acquired from outside the simulated population due to human or vector mobility). The external force-of-infection was set to zero in half of the simulation runs, to mimic communities where transmission is independent of imported infections. In the other runs, it was set to a low value, varying between runs but constant over time to mimic communities where low endemicity is stabilized by incoming infection from surrounding areas.

We then checked whether the model-predicted association between mf and CFA prevalences matched to village-level data at baseline and after the first, second and third annual treatment. We further assessed whether the model adequately captures time trends in infection. Lastly, we compared model-predicted and observed age-patterns in infection for all measurements moment, aggregating data from different communities by country. A more detailed description of the data and validation methods is provided in section 2 in S1 Supplement.

### Predictive values of Mf and CFA prevalence for elimination and resurgence

We assessed how well the eventual occurrence of elimination of transmission can be predicted based on the Mf or CFA prevalence one year after the last treatment round. We simulated four MDA scenarios with either six or eight annual rounds of MDA with ivermectin plus albendazole, and either 60% or 80% treatment coverage of the total population (with treatment restricted to individuals aged 5 years or older). Bed net use was not considered in these simulations, as we were interested in the dynamics of resurgence and elimination in the absence of bed nets.

This analysis was done for settings with an average population size of about 1000 individuals and a moderate to high baseline Mf prevalence (i.e. between 20% and 40%). We only considered settings without importation of infection from surrounding areas (external force of infection = 0), so that elimination outcomes were not influenced by the presence or absence of importation from surrounding areas. Between runs we varied the monthly biting rate and the shape of the gamma distribution describing variation in exposure between individuals. Parameters were sampled from the parameter space presented in Fig A in S1 Supplement (left panel) and were accepted when the resulting pre-control Mf prevalence fell into one of the 1%-width bins between 20% and 40%; the model was run until we had 100 parameter combinations per bin, resulting in 2000 runs per scenario.

For each run, we recorded predicted time trends in Mf and CFA prevalence in the population aged 5 or 15 years and above, as well as the CFA prevalence among 6-7-year-old children, from a year before the first treatment until 50 years after the last treatment round. Elimination was defined as zero Mf prevalence in the 5+ population 50 years after the last treatment round. To assess to which extent the eventual occurrence of elimination can be predicted based on Mf and CFA prevalence surveys done 1 year after the last treatment, we calculate receiver operating characteristic (ROC) curves for a range of prevalence thresholds, assuming that all individuals per age group are examined. For the calculation of PPV and NPV values, we assumed that a random binomial sample of 200 individuals in the respective age group was surveyed (sampled with replacement); as the number of 6-7-year olds is much lower than this, this implies that nearly all individuals in this age group are included in the survey. This sampling was repeated 100 times to smooth the resulting predictions. Stop-MDA thresholds were estimated at a PPV of 95%. A sample size of 200 was chosen, assuming that this would be a practically feasible number, but alternative values were also used in a sensitivity analysis.

Lastly, we assessed how assumptions regarding the rate of Mf production per worm (which influences the association between Mf density and the underlying number of worms) affect the predicted elimination thresholds. The Mf productivity rate was doubled or halved from 0·58 (estimated previously by fitting model to data)[17] to 1·16 or 0·29 Mf/μL blood per female worm per month); to counter the impact on transmission intensity, we simultaneously halved or doubled the mosquito biting rates (see Fig B in S1 Supplement).

### PRIME-NTD principles

In S1 Supplement section 3, we describe our adherence to the five principles of the NTD Modelling Consortium on good practice for policy-relevant modelling [19].

## Results

### Comparison of model-predicted and observed Mf-CFA prevalence associations

**Fig 1:**
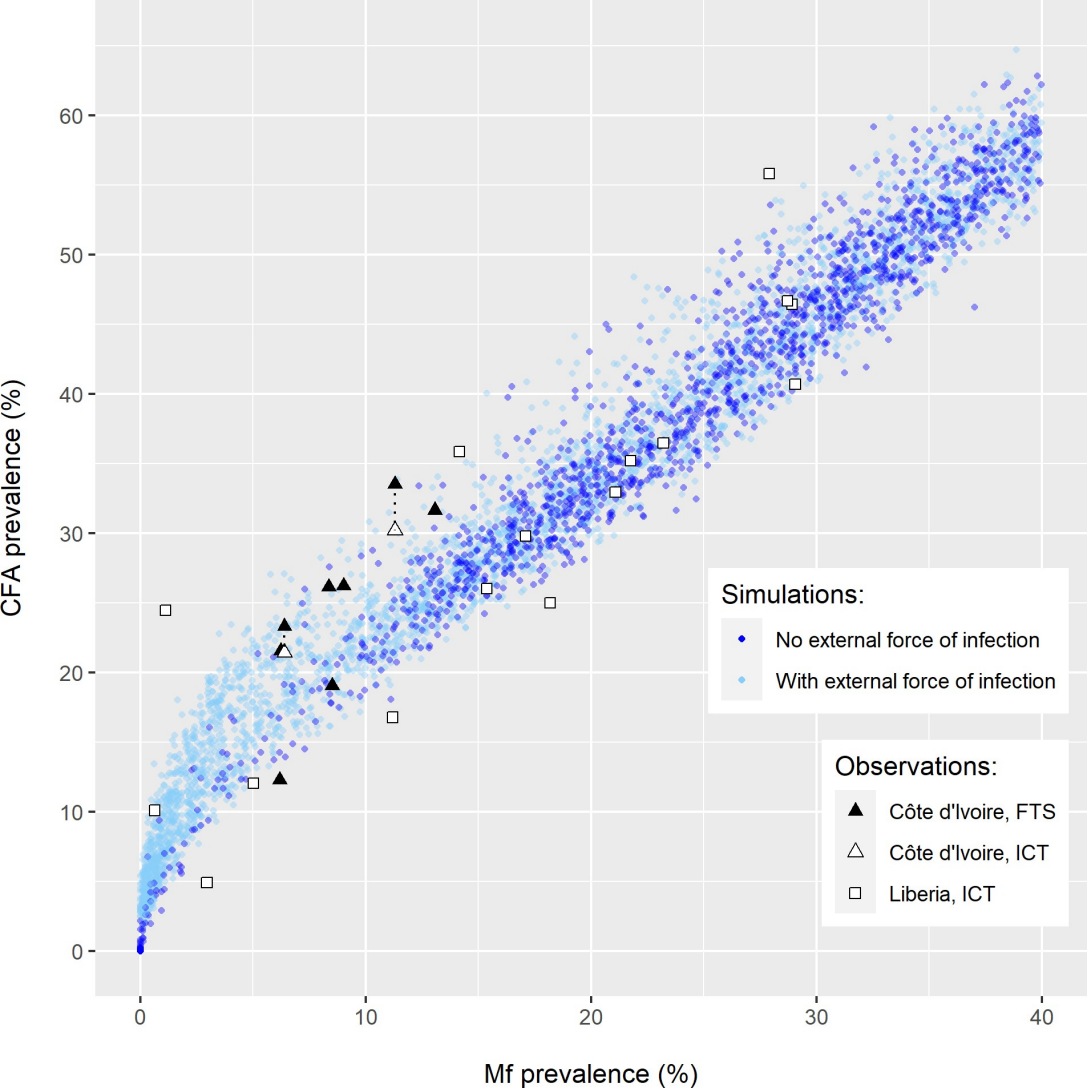
Comparison of the model-predicted and observed pre-treatment association between microfilaraemia (Mf) and circulating filarial antigenaemia (CFA) prevalence at community-level. Observed data are from villages in the annual and biannual treatment arms in Côte d’Ivoire (triangles) and Liberia (squares), with CFA prevalences assessed using either the filarial test strip (FTS, black) or the immunochromatographic card test (ICT, white). FTS- and ICT-based observations made in the same community are connected via black dotted lines. Model predictions (dots) are shown for settings without (dark blue) and with (light blue) an external force of infection.

Fig 1 shows that model-predicted Mf-CFA prevalence combinations before the introduction of MDA matched well to observed data. The FTS-based observed CFA prevalences from Côte d’Ivoire were relatively high, considering the corresponding Mf prevalences, but could be matched by the model by including importation of infection from surrounding areas. The ICT-based observed CFA prevalences for Liberia were often relatively low for given Mf prevalences and could often be captured without assuming infection importation.

**Fig 2:**
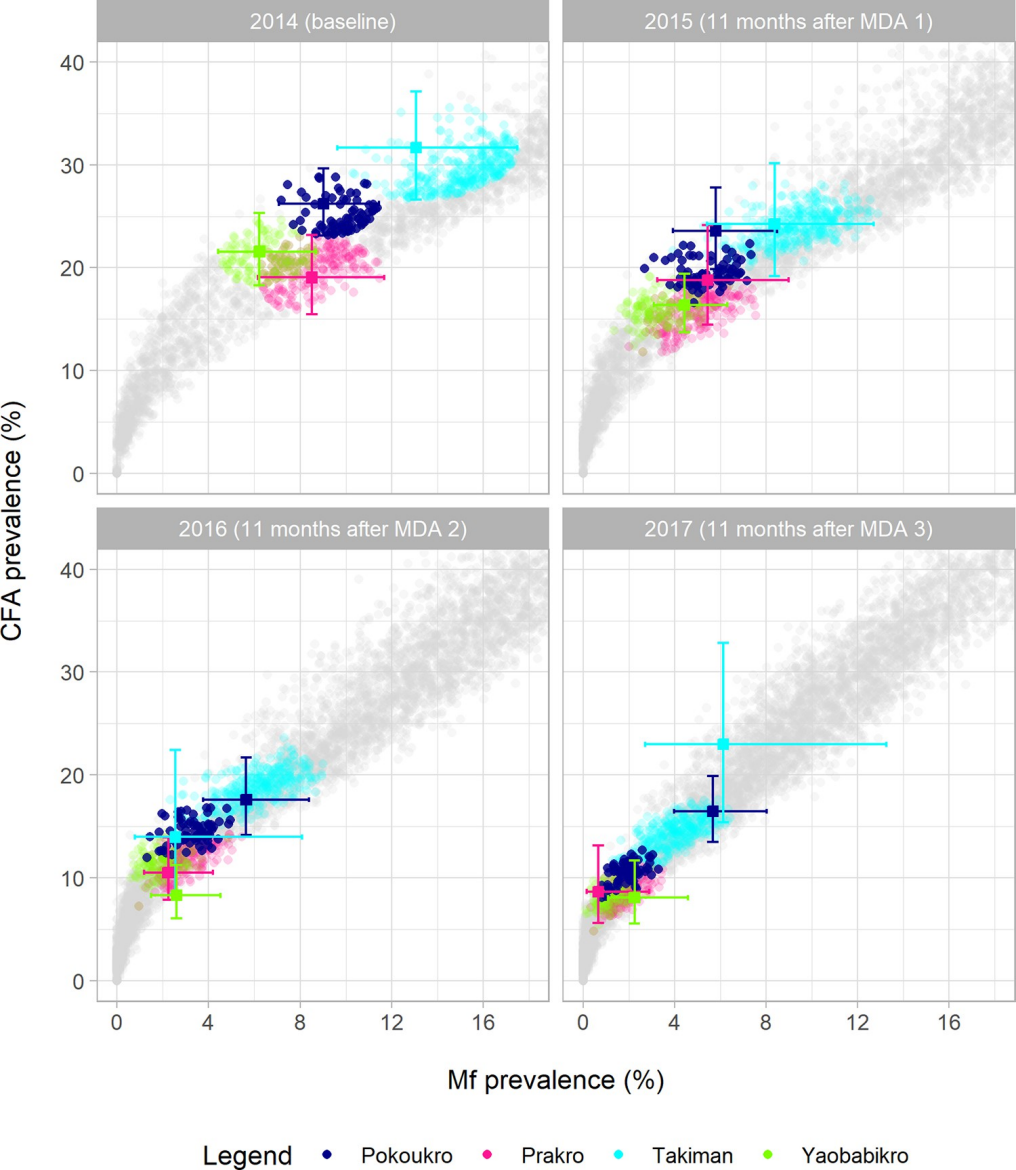
Observed and simulated prevalence of microfilaraemia (Mf) and circulating filarial antigenaemia (CFA, measured by filarial test strip) for Côte d’Ivoire, at baseline (2014, before the first treatment round) and in 2015, 2016, and 2017 (i.e. 11 months after the first, second and third annual MDA rounds). Observations are shown as coloured markers with 95% confidence intervals. Model predictions are shown as small dots. Simulation results from runs matched to specific villages at baseline are shown in the colour of that village, and all other runs are shown in grey. A run was considered a match if the predicted Mf-CFA prevalence combination at baseline fell within the ellipse drawn around the observed MF-CFA prevalence combination based on the 95% confidence intervals. For both the model and the observed data, crude prevalence estimates are presented in the figures (i.e. not age-standardized). The MDA coverage was assumed to be 65% of the total population per round in the simulation runs. See Table B in S1 Supplement for details about the simulated scenario and see Fig C in S1 Supplement for a similar figure for Liberia.

Fig 2 shows that the model also adequately captured the association between community-level Mf and CFA prevalence in Côte d’Ivoire during MDA (11 months after the first, second and third treatment round). However, the predicted Mf and CFA prevalence levels per village for the follow-up moment did not always match the observations. Deviations can result from sampling variation in the observed data (sometimes leading to erratic patterns in the data that are not mimicked by the model) or from a mismatch between assumed and actual coverage of MDA (true coverage is unknown and can vary over time and between communities). Fig C in S1 Supplement presents similar results for Liberia, which are somewhat more difficult to interpret due to a switch in CFA diagnostic from ICT at baseline to FTS in later surveys, with the latter resulting in somewhat higher CFA prevalences (indicated by observations from communities were both tests were used in parallel). At baseline, the observed prevalences from Liberia were usually located within the band of predicted values, whereas they were close to the upper edge of the model predictions band or even above it at the three follow-up moments. This was most extreme after the third treatment round, when Mf-prevalence was zero in all communities but CFA prevalence up to 13% were observed. Various factors could contribute to the deviation between model predictions and observations, including uncertainty about MDA coverage and treatment effects on adult worms. A lag in antigen clearance after worm killing could theoretically also explain some of these results. However, due to the long time-interval between treatment and the follow-up surveys, this is not the most likely explanation. Also, if this plays a role, we would expect it to be visible in both countries and not only in Liberia. The observed reductions in Mf and CFA prevalence were larger than predicted by the model, for most villages, again suggesting a mismatch between assumed and actual coverage of MDA. Still, for both Liberia and Côte d’Ivoire the observed Mf and CFA prevalence combinations were all on or near the model-predicted associations.

Figs D and E in S1 Supplement show that the model also captured the age-patterns in Mf and CFA prevalence, including the considerably lower prevalence levels in children compared to adults.

### Predictive performance of MF- and CFA-based stop-MDA thresholds

**Fig 3:**
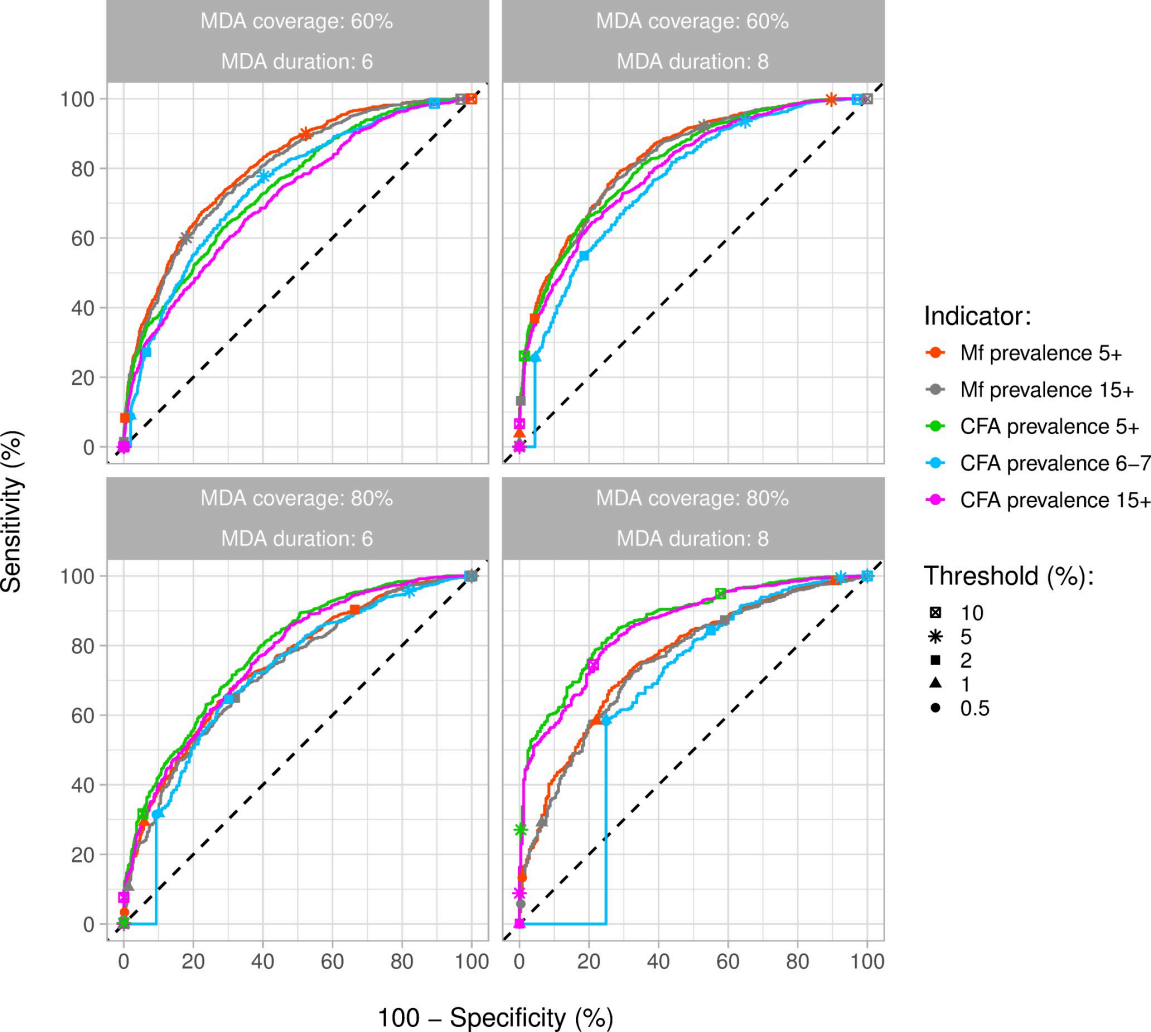
Receiver-operator characteristic (ROC) curves for predicting the eventual occurrence of elimination of transmission under different MDA scenarios, based on the simulated microfilaraemia (Mf) or circulating filarial antigenaemia (CFA) prevalence 1 year after the last treatment round. Different lines show the predictive performance of the Mf and CFA prevalence assessed in all people aged 5 or 15 years and above (Mf: red and grey; CFA: green and pink) and the CFA prevalence in all 6-7-year-old children (blue). Sensitivity is the percentage of simulation runs ending in elimination that are correctly identified based on Mf or CFA prevalence below a range of thresholds (see legend). Similarly, 100%-specificity is the percentage of simulation runs that is falsely classified as having achieved elimination, out of all runs that did not result in elimination, resulting in premature stopping of MDA. The optimal situation is in the upper left corner of the panels (100% sensitivity and 100% specificity). Results are based on 2000 simulations per scenario, with baseline Mf prevalence varying between 20% and 40%.

Model-predicted trends in Mf and CFA prevalence during and after MDA (six or eight rounds, 60% of 80% coverage) are shown in Figs F and G in S1 Supplement for settings with baseline Mf prevalence between 20%-30% and 30%-40% in the population aged 5 years and above. Longer duration of MDA and higher coverage resulted in lower Mf and CFA prevalence one year after the last treatment and higher probability of elimination. The ROC curves in Fig 3 show how well the eventual occurrence of elimination could be predicted from Mf and CFA prevalence one year post-MDA, for different age groups (assuming that all individuals per age group are included in the survey) and for a range of different threshold values. Adopting a higher threshold value results in a higher sensitivity (i.e. a higher proportion of runs ending in elimination correctly classified as such, based on a prevalence below the chosen threshold), but also a lower specificity (i.e. a lower proportion of runs ending in resurgence correctly classified as such, based on a prevalence above the chosen threshold). This lower specificity means a higher probability of falsely declaring elimination and prematurely stopping MDA, the most important adverse outcome. In the scenario with six treatment rounds with 60% coverage, the Mf prevalence in the 5+ or 15+ population seemed to be a slightly better indicator of elimination than CFA prevalence in any of the age groups considered. However, the overall probability of elimination was still low in this scenario (43% of all runs). The predictive value of CFA prevalence in the 5+ or 15+ population increased with longer MDA and higher coverage, and hence higher elimination probability. The CFA prevalence in the 5+ and 15+ population clearly were the best predictors in the scenario with eight treatment rounds and 80% coverage and the CFA prevalence among 6-7-year olds was the worst predictor, independently of the threshold considered. In this scenario, about 87% of runs resulted in elimination. These patterns remained when the results were further stratified by endemicity, based on baseline Mf prevalence in the 5+ population (Fig H in S1 Supplement).

**Fig 4:**
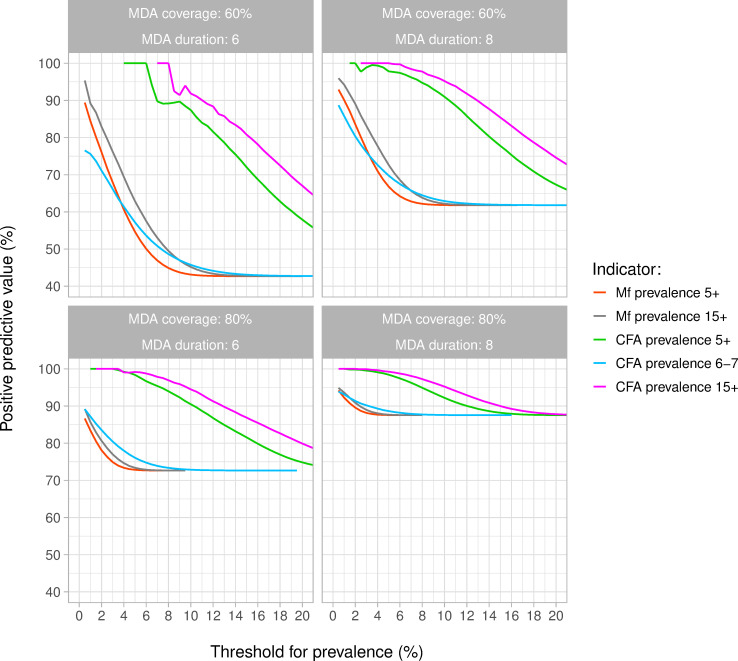
Probability of achieving elimination within 50 years after the last round of MDA, if the 1-year post MDA prevalence was below a given threshold (i.e. the positive predictive value of using this threshold). Different lines show the predictive performance of the Mf and CFA prevalence in a sample of 200 individuals taken from the 5+ or 15+ population (MF: red and grey; CFA: green and pink) and the CFA prevalence in a sample of 200 6-7-year-old children (blue). Results are based on 2000 simulations per scenario, with baseline Mf prevalence varying between 20% and 40%. The PPV curves based on CFA prevalence in the 5+ or 15+ population are somewhat erratic at low prevalences in the panel for settings that had 6 rounds of MDA with 60% coverage. This is explained by the fact that only few simulation runs ended with such low CFA prevalences in this treatment scenario (i.e. the denominator for calculating the PPV). If one of these few runs ended in resurgence, it causes relatively large fluctuations in the estimated PPV.

Fig 4 shows per indicator how the PPV (probability of achieving elimination within 50 years after the last round of MDA if the 1-year post MDA prevalence in a sample of 200 individuals per age group was below a given threshold) depended on the chosen threshold. The PPV never reached 100% for CFA prevalence among 6-7-year olds and Mf prevalence in samples of the 5+ or 15+ population. This means that there was still a risk of resurgence, even when the simulated CFA or Mf prevalence was zero. A PPV value of 100% could only be achieved with the stop criterion based on CFA prevalence in the 5+ or 15+ population. The threshold prevalence associated with a 95% PPV was about 0·5% for the Mf prevalence in the 15+ population, if reached at all. The thresholds based on CFA prevalence in the 5+ and 15+ population were much higher, i.e. 8% and 10% respectively. The corresponding NPV (probability of recrudescence if prevalence was above the threshold) declined from nearly 60% in the scenario with six MDA rounds with 60% coverage to about 25% in the scenario with eight MDA rounds with 80% coverage (Fig I in S1 Supplement). The PPV of the suggested threshold was slightly higher among runs with 30–40% baseline Mf prevalence than in runs with 20–30% baseline Mf prevalence (Fig J in S1 Supplement).

The PPV for a given Mf or CFA prevalence threshold increases with the number of people sampled and thresholds associated with 95% PPV shift to higher values (Fig K in S1 Supplement). Mf prevalence thresholds still remain low, and the PPV for CFA prevalence among 6-7-year olds never exceeds 90%. For a given sample size, measuring CFA or Mf in the 15+ population results in slightly higher PPV and higher threshold values than measuring CFA prevalence in the 5+ population, because the prevalence of infection is lower in 5–15 year old children. Halving or doubling Mf-productivity (while doubling or halving the biting rate) had hardly any impact on the pre-control Mf-CFA prevalence association and the positive predictive value of the suggested thresholds (Figs L and M in S1 Supplement).

## Discussion

We showed that the eventual occurrence of elimination cannot be predicted with sufficient certainty from the CFA prevalence measured among 6-7-year-old children, irrespective of the number tested, nor from the Mf prevalence measured in a sample of up to 200 individuals from the 5+ or 15+ population. While the predictive value of an Mf-based stop-MDA threshold would improve if based on a full-population survey, thresholds remain low, requiring high (and more costly) sampling sizes. Elimination can be predicted with much more certainty based on CFA prevalence in the 5+ or 15+ population, which has the added advantage of considerably higher thresholds and thereby smaller sample sizes needed. Focusing on the 15+ age group may be preferred, as a high PPV can be achieved with higher thresholds and thus lower sample sizes than when focussing on the entire 5+ population. It has the added advantage that 5–14 year old children don’t need to be tested, which could make the surveys more acceptable to the community and easier to execute. A CFA prevalence of 10% in this population subgroup corresponds to a PPV of 95% or higher, and this threshold is robust for endemicity level and number of MDA rounds.

The proposed CFA threshold, chosen for its high PPV (≥95%), is associated with a relatively low NPV (i.e. the probability of resurgence if the prevalence is above the threshold), with NPV values between 25% and 60% depending on the treatment scenario. We consider this acceptable as the consequence of a false negative outcome (continuing MDA for too long at the cost of wasting drugs and other resources) is less of a problem than a false positive result (stopping MDA too early and having to reinstate the MDA infrastructure).

To reproduce the pre- and post-MDA Mf-CFA prevalence association with our model, it was sufficient to assume that CFA positivity occurs when an individual has at least one mature worm, even though village-specific time trends in Mf and CFA prevalence were not always matched exactly. Some unexplained variation remains in the data, which may be due to sampling variation or other factors not captured in the model. For example, direct comparison of the ICT card test and the Filarial Test Strip (FTS) for measuring antigenaemia suggested that the latter is more sensitive, especially in low endemic settings [13]. Accounting for this would bring the model closer to the relatively low ICT-based CFA prevalences observed in Liberia (Fig 1). Also, accounting for the impact of ivermectin MDA for onchocerciasis prior to the trial onset in Côte d’Ivoire, could help to reproduce observations with relatively low Mf prevalence and high CFA prevalence, as ivermectin causes a strong reduction in Mf density but does not kill adult worms [20,21]. Further refinement of the diagnostic model, e.g. to capture temporal dynamics of antigenaemia density after worm-death or a possible differential contribution of male and female worms to antigenaemia density, requires more-detailed individual-level infection and treatment data. More detailed modelling of antigenaemia decay rates would likely have little impact on model predicted antigenaemia prevalences 12 months after treatment, on which our threshold estimates were based. However, even If CFA tests remain positive that long after treatment-induced death of the last worm, this would not change the main findings, but would only result in slightly higher threshold values.

Uncertainty is inherent to modelling, e.g. concerning the values of model parameters or model structure. We used the LYMFASIM model, which was quantified for *Anopheles*-transmitted bancroftian filariasis in Africa and was validated in this study against data from West Africa. It is reassuring that our PPV and NPV estimates for CFA prevalence among 6-7-year olds are fairly similar to those estimated with another simulation model [22]. Their estimates for Malindi (a Kenyan village with baseline endemicity within our simulated range) are comparable to our estimates from the most intensive MDA scenario (with eight MDA rounds and 80% coverage), both with respect to the overall probability of elimination and the PPV of different infection indicators. We anticipated that our predictions would be influenced by assumptions about the ratio of Mf to adult worm density. We explored the impact of using a different Mf productivity rate, but found that doubling or halving this value (and thus halving and doubling corresponding worm burdens) did not change the overall conclusions regarding the relative performance of different indicators for stopping. Still, in-depth analysis of individual-level Mf and CFA positivity or density data could help to quantify such uncertain parameters to further improve the accuracy of our model [23].

WHO chose to assess infection in 6-7-year-old children, as any infection in this age group must be recently acquired, meaning that transmission has not been completely interrupted [6]. Focussing on this age group is also practical, as it allows for school-based sampling. The 2% CFA prevalence threshold was assumed to be “a conservative proxy for an Mf prevalence of <1%”, considering the higher sensitivity of CFA tests for detecting infection [6]. However, our study clearly demonstrates that CFA prevalence in this age group is not the most informative predictor of the eventual achievement of elimination. As 6-7-year olds constitute a small proportion of the population and are less exposed than older individuals, even a zero prevalence in this group does not preclude eventual resurgence of LF in the population (demonstrated by the model-estimated PPV values in this age group never exceeding 90%).

Our simulations were specific for African areas where LF is transmitted by *Anopheles*-species and ivermectin plus albendazole is the MDA treatment regimen. Our thresholds are not valid for areas, where LF is transmitted by different vector species (e.g. *Culex* or *Aedes*). Especially the *Aedes*-vectors are known to be highly efficient, which led WHO to propose lower stop-MDA thresholds for areas with *Aedes* transmission. The employed treatment regimen can also influence the post-MDA CFA-Mf association and hence the CFA-thresholds. However, our conclusions about the insufficient sensitivity of TAS in 6-7-year-old children are generalizable. Indeed, concerns about the reliance on measures in such young children were voiced before, when evidence of persistent LF transmission was found after passing TAS and stopping MDA, e.g. in Sri Lanka and American Samoa [24–27]. Detailed studies in these countries carried out several years after passing TAS and stopping MDA, confirmed that CFA prevalence in such young children was considerably lower than the prevalence among older individuals or adults only [11,28]. Moreover, these studies demonstrated that community-based TAS is feasible, although it is logistically more difficult, takes more time and is more expensive.

Our quantitative CFA prevalence threshold estimates (10% in 15+) should be interpreted with caution, also for the African context. They are based on modelling and our long-term elimination predictions remain to be validated. These quantitative thresholds apply to communities with moderate to high baseline Mf prevalence (between 20% and 40%) and under the specified transmission and treatment conditions. In reality there may be more variation in transmission conditions, treatment history, or age-infection patterns, which can influence the safe threshold values. For example, lower thresholds may be needed in settings where low pre-MDA Mf prevalence is stabilized by continued imported infection from surrounding areas or strong aggregation of exposure, as was shown in other theoretical studies for filarial infections [22,29,30]. To obtain more certainty about the stop-MDA decision, presence of Mf can be assessed in CFA positives to ensure absence of a significant Mf reservoir for transmission. This is feasible, as night blood sampling and processing would be required for a limited number of people. If stop-MDA decisions are made for larger geographical areas, encompassing multiple communities with varying baseline endemicities, thresholds should be adjusted to account for heterogeneity within the area. This is most obvious when the area includes non-endemic communities, which cause dilution of infection levels for the endemic part of the area, requiring lower threshold levels.

We conclude that the currently used CFA prevalence in 6–7 year-old children is a poor indicator for stopping decisions in LF control by MDA if elimination of transmission is the eventual goal. We recommend using CFA prevalence of the 15+ population at a threshold of 10% (with Mf follow-up) as critical threshold for stopping MDA in LF elimination programmes where treatment has been provided for at least 5 or 6 years with adequate coverage, for application at the level of communities or somewhat larger areas with relatively homogeneous transmission conditions.

## Supporting information

S1 Supplement PDF file with supplementary information to the manuscript. The document has the following sections: section 1) LYMFASIM: model and parameter values; section 2) Validation of model-predicted CFA prevalence levels; section 3) PRIME-NTD table: Policy-Relevant Items for Reporting Models in Epidemiology of Neglected Tropical Diseases; section 4) Detailed results.
(PDF)
