# Supplementary material for: Comparing antigenaemia- and microfilaraemia as criteria for stopping decisions in lymphatic filariasis elimination programmes in Africa
Source: PLoS Negl Trop Dis. 2022 Dec 12;16(12):e0010953. doi: 10.1371/journal.pntd.0010953 (PMC9779720; doi:10.1371/journal.pntd.0010953)
Supplement: S1 Supplement — The document has the following sections: section 1) LYMFASIM: model and parameter values; section 2) Validation of model-predicted CFA prevalence levels; section 3) PRIME-NTD table: Policy-Relevant Items for Reporting Models in Epidemiology of Neglected Tropical Diseases; section 4) Detailed results. (PDF) [file pntd.0010953.s001.pdf]

S1 Supplement

Comparing antigenaemia- and microfilaraemia as criteria for stopping decisions in lymphatic filariasis elimination programmes in Africa

Wilma A. Stolk[1*], Luc E. Coffeng[1], Fatorma K. Bolay[2†], Obiora A. Eneanya[3], Peter U. Fischer[3], T. Déirdre Hollingsworth[4], Benjamin G. Koudou[5,6], Aboulaye Méité[7], Edwin Michael[8], Joaquin M. Prada[9], Rocio M. Caja Rivera[8], Swarnali Sharma[10,11], Panayiota Touloupou[12,13], Gary J. Weil[3], Sake J. de Vlas[1]

Affiliations

[1] Department of Public Health, Erasmus MC, University Medical Center Rotterdam, Rotterdam, The Netherlands

[2] National Public Health Institute of Liberia (NPHIL), Monrovia, Liberia

[3] Infectious Diseases Division, Department of Medicine, Washington University School of Medicine, St. Louis, Missouri, USA

[4] Big Data Institute, Li Ka Shing Centre for Health Information and Discovery, University of Oxford, UK

[5] Centre Suisse de Recherches Scientifiques en Côte d'Ivoire, Abidjan, Abidjan, Côte d'Ivoire

[6] Laboratoire de Cytologie et Biologie Animale, UFR Science de la Nature, Université Nangui Abrogoua Abidjan, Abidjan, Côte d'Ivoire

[7] Programme National de Lutte contre les Maladies Tropicales Négligées à Chimiothérapie Préventive, Abidjan, Côte d'Ivoire

[8] Center for Global Health Infectious Disease Research, University of South Florida, Tampa, USA

[9] Faculty of Health and Medical Sciences, University of Surrey, UK

[10] Department of Biological Sciences, University of Notre Dame, Notre Dame, South Bend, IN, USA

[11] Christian Medical College, IDA Scudder Rd, Vellore, Tamil Nadu 632004, India

[12] Department of Statistics, University of Warwick, UK

[13] School of Mathematics, University of Birmingham, UK

[†] Deceased

Keywords: lymphatic filariasis, elimination, mass drug administration, mathematical modelling, ivermectin, albendazole, endpoints

Running title: Stopping decisions in lymphatic filariasis elimination programmes

* *Corresponding author:* Wilma A. Stolk, PhD; Department of Public Health, Erasmus MC, University Medical Center Rotterdam, PO Box 2040, 3000 CA Rotterdam, The Netherlands. Phone: +31 10 704 3730; Email: w.stolk@erasmusmc.nl

Contents

Section 1. LYMFASIM: model and parameter values 3
1.1 Model version and availability 3
1.2 Model description 3
1.3 Model parameters and their values 4
Section 2. Validation of model-predicted CFA prevalence levels 10
2.1 Data 10
2.2 Methods 11
Section 3. PRIME-NTD table: Policy-Relevant Items for Reporting Models in Epidemiology of Neglected Tropical Diseases 13
Section 4. Detailed results 15
References 26

Section 1. LYMFASIM: model and parameter values

1.1 Model version and availability

Model version: wormsim version 2.58Ap59.

Programme and source code availability: LYMFASIM was originally developed as a standalone computer programme [1], but is now incorporated as a disease-specific variant within WORMSIM, a generalized framework for modelling transmission and control of helminth infections in humans. A formal description of WORMSIM has been provided elsewhere for version v2.58Ap9 [2]. The programme and source code are available at gitlab: https://gitlab.com/erasmusmc-public-health/wormsim.previous.versions

1.2 Model description

The LYMFASIM model has been described elsewhere [1,3] and it has been applied to support decision making on control and elimination of lymphatic filariasis in different settings [3-6]. We restrict here to a brief description.

LYMFASIM is a stochastic individual-based model for simulating lymphatic filariasis (LF) transmission and control in a closed, dynamic population, typically representing the population from a village or small town. Each human individual is simulated separately. The population composition changes over time, because of birth, death and emigration (removal) of individuals from the population. The infection status (number of adult worms for each sex, mf density) for each individual in the population is tracked over time. Exposure to mosquito bites is assumed to vary between individuals, driven by age and sex patterns in exposure as well as by stochastic variation between individuals. As a result, infection levels vary between individuals. Female adult worms produce microfilariae (mf) when at least one male worms is present in the same host (polygamous mating). The uptake and transmission of infection between hosts are simulated deterministically, accounting for the variation in exposure between individuals.

LYMFASIM can be used to simulate the effect of interventions (e.g. mass drug administration, integrated vector management, bednet use) on transmission and morbidity, taking account of the human demography and the complexities of helminth transmission. Mass drug administration (MDA) is simulated by specifying the year and month in which treatment takes place, the efficacy of the applied treatment regimen, the achieved coverage level, and compliance patterns. Systematic non-participation is simulated by assuming that a fraction of the population never participates in MDA (e.g. systematic refusal, related to chronic illness). In addition, LYMFASIM allows the relative compliance to vary between age and sex groups; this mechanism captures transient contra-indications for MDA (e.g. exclusion of young children and pregnant women) and other age- and sex-related behavioural factors driving

participation in MDA. Lastly, each individual has a personal inclination to participate in MDA, which is considered as a lifelong property. A stochastic process eventually defines per individual whether he is treated in a given round, depending on the calculated probability.
The impact of bednets is simulated by assuming that a random fraction of the population is using bednets (here, this fraction equals the bednet coverage in the population) and that the mosquito biting rate among bednet-users is 97% lower than expected without bednets.

1.3 Model parameters and their values

We previously derived model quantifications for simulating transmission of bancroftian filariasis by *Anopheles* species in Africa[7], accounting for the age-structure of the human population and density dependence in the L3 yield from a blood meal in mosquitoes. Acquired immunity was not considered to play a role in the Africa model [7]. Parameter values relating to human demography, human exposure to mosquitoes, the parasite life cycle and transmission, and treatment efficacy are listed in Table A below. Assumptions and parameters related to control strategies and treatment efficacy are listed in Table B (section 2 of this supplement).

Table A. LYMFASIM input: probability distributions, functions and parameter values

Parameter description (symbol)	Model variant for Africa		Source / remarks
**Human demography**			
Cumulative survival (F(a)), by age	Age	Survival	Fixed, as in [7]
	0	1	
	5	0.804	
	15	0.78	
	20	0.755	
	25	0.73	
	30	0.707	
	35	0.654	
	40	0.605	
	45	0.56	
	50	0.506	
	60	0.407	
	70	0.255	
	80	0.051	
	99	0	
Fertility rate per woman (R(a)), by age	Age	Fertility rate	Fixed, as in [7]
	0	0	
	5	0	
	15	0	
	20	0.116	
	25	0.230	
	30	0.245	
	35	0.207	
	40	0.147	

Parameter description (symbol)	Model variant for Africa	Source / remarks
	45 0.077<br>50 0.031<br>60 0<br>70 0<br>80 0<br>99 0	
Initial population	Age Male/females<br>5 42/42<br>15 63/63<br>20 26/26<br>25 22/22<br>30 20/20<br>35 17/17<br>40 14/14<br>45 11/11<br>50 9/9<br>60 14/14<br>70 9/9<br>80 3/3<br>99 1/1	Assumed
Maximum population size	Varied between simulations, as described below	
Proportion removed when maximum population size is reached	5%	Assumed
**<u>Transmission initialization</u>**		
External force-of-infection at start of burn-in period	2	Assumed
Duration of external force-of-infection at start of burn-in period	2 years	Assumed
Duration of warming up period	156	Assumed
**<u>Transmission dynamics after initalization</u>**		
Average mosquito biting rate for adult men (mbr)	Varied between simulations as described below	Assumed
Seasonal variation	No seasonal variation (monthly biting rate is the same in all months)	Assumed
Relative biting rate (multiplier of mbr that can be used scale this seasonal pattern to some desired level)	1	
Variation in exposure by age (no difference assumed between sexes)	0 at birth, linearly increasing to reach 1 at the age of 20 and constant at 1 from this age onwards	Previously estimated by fitting to data[3]; slightly adjusted for Africa[7]

Parameter description (symbol)	Model variant for Africa	Source / remarks
Probability distribution describing variation in the individual exposure index (Ei), due to personal factors (fixed through life) given age and sex	Gamma distribution with mean 1.0; shape (=rate) is varied as described below	Gamma distribution is assumed;
External force of infection	0	
**Parasite dynamics within host**		
Success ratio (sr)	0.00088	Previously estimated by fitting to data[7]
Anti-L3 immunity	Not considered in this study (strength of immunological memory for anti-L3 immunity = 0; duration of immunological memory for anti-L3 immunity = 0)	Assumed, as justified in [7]
Anti-fecundity immunity:	Not considered in this study (strength of immunological memory for anti-fecundity immunity = 0; duration of immunological memory for anti-fecundity immunity = 0)	Assumed, as justified in [7]
Average worm lifespan (Tl)	10 years on average; varied according to a Weibull distribution with shape 2	Previously estimated by fitting to data[3]
Duration of immature stage of the parasite in human host (Ti)	Constant, 8 months	Fixed [8]
No. of Mf produced per female parasite per month per 20 ml peripheral blood in the absence of immune reactions and in the presence of at least 1 male worm (r0)	0.58	Previously estimated by fitting to data[3]
Monthly survival of the microfilariae, fraction (s)	0.9	Fixed, based on[9]
Association between worm age and mf production rate	Mf production independent of worm age	Assumed
Polygamy (all female worms produce mf in the presence of at least one male worm)	Yes (male potential 1000)	
Mating cycle (number of months a female can produce mf with one insemination)	1	Assumed
**Uptake of infection by the vector**		
Functional relationship	$L3 = a(1 - \exp(-(bM)^c))$	

Parameter description (symbol)	Model variant for Africa	Source / remarks
	a 1.666<br>b 0.027<br>c 1.514	Previously estimated by fitting model to data [7]
Transmission probability (v), fraction of the L3 larvae, resulting from a single blood meal, that is released by a mosquito	0.1	Fixed, as in [3]
**<u>Surveillance</u>**		
Timing of surveys	See scenario description	
Volume of blood examined for mf	60 µL	Data
Variability in observed number of mf in one 20 µl blood smear	Negative binomial distribution with k=0.33	Previously estimated for 20 µL blood by fitting to data[3], assumed for Africa
Variation between worms in their contribution to measured mf count (dispersal factor)	Constant (no variation)	Assumed
**<u>Morbidity & excess mortality due to disease</u>**		
Not considered		
**<u>MDA and vector control</u>**		
Timing and coverage of treatment	See scenario description below	
Fraction of the population never participating in treatment (= proportion systematic non-compliers)	0.05	
Minimum age for treatment	5 years	
Relative compliance by age and sex	Age / Males / Females<br>0-4 / 0 / 0<br>5-9 / 0.75 / 0.75<br>10-14 / 0.80 / 0.70<br>15-19 / 0.80 / 0.74<br>20-29 / 0.70 / 0.65<br>30-49 / 0.75 / 0.70<br>50-59 / 0.75 / 0.70<br>60-99 / 0.80 / 0.75	Assumed based on unpublished data from the Onchocerciasis Control Programme
Vector control	See scenario description below	
**<u>Efficacy of ivermectin + albendazole, per treatment</u>**		
Fraction malabsorption	0	Assumed
Permanent reduction in female worm reproductive capacity	0	
Average duration of temporary reduction in female reproductive capacity in years (parameter determining shape of the recovery function)	0.75 (1000)	

Parameter description (symbol)	Model variant for Africa	Source / remarks
Fraction of adult worms killed	0.35	
Treatment effect variability	NA (assumed constant, 1)	Assumed
Fraction of microfilariae surviving treatment	Constant, 0.01	

The relative montly biting rate (mbr) and the shape (=rate) parameter (k) of the Gamma distribution describing exposure heterogeneity in the simulated population were varied according to the density plot in Figure A below, in order to generate simulations across a wide range of mf prevalences at baseline, measured in the population aged 5 and above.

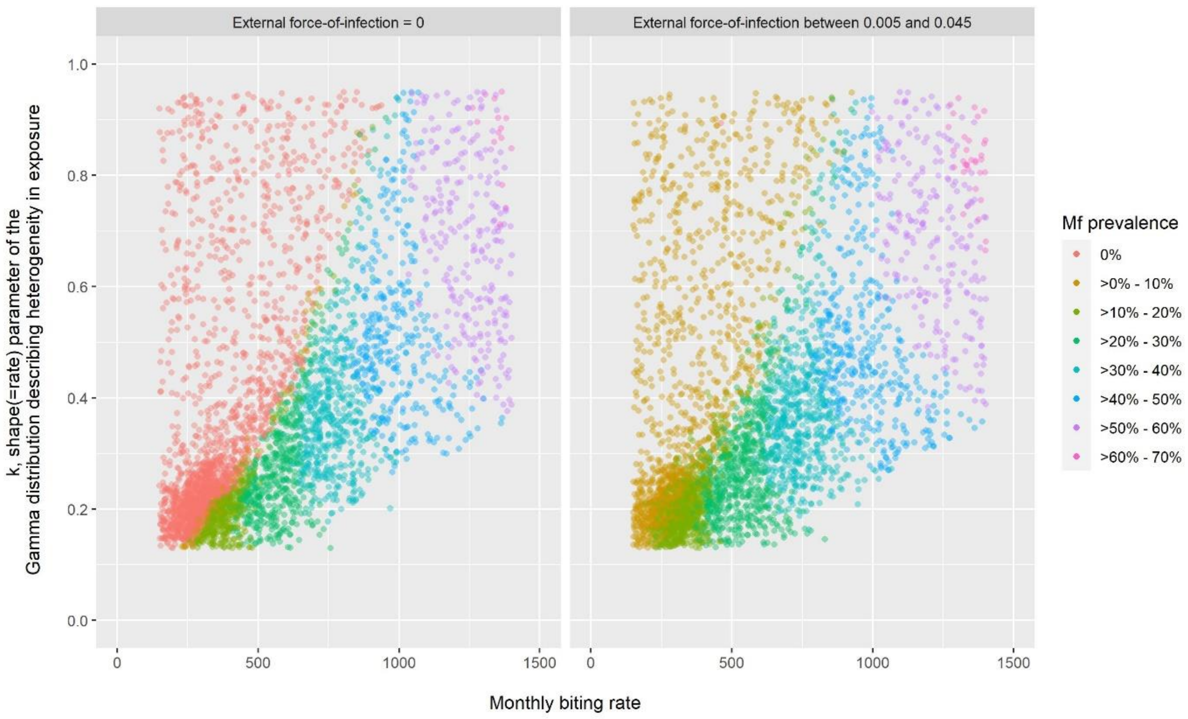

Figure A. Density plot illustrating the parameter space, showing simulated combinations of parameters for the monthly biting rate, the shape (=rate) of the gamma distribution describing exposure heterogeneity in the population (k), and the external force of infection, and the resulting baseline mf prevalences in 2013 for Côte d'Ivoire. The model accounts for low-coverage bednet use in the study area since 2006.

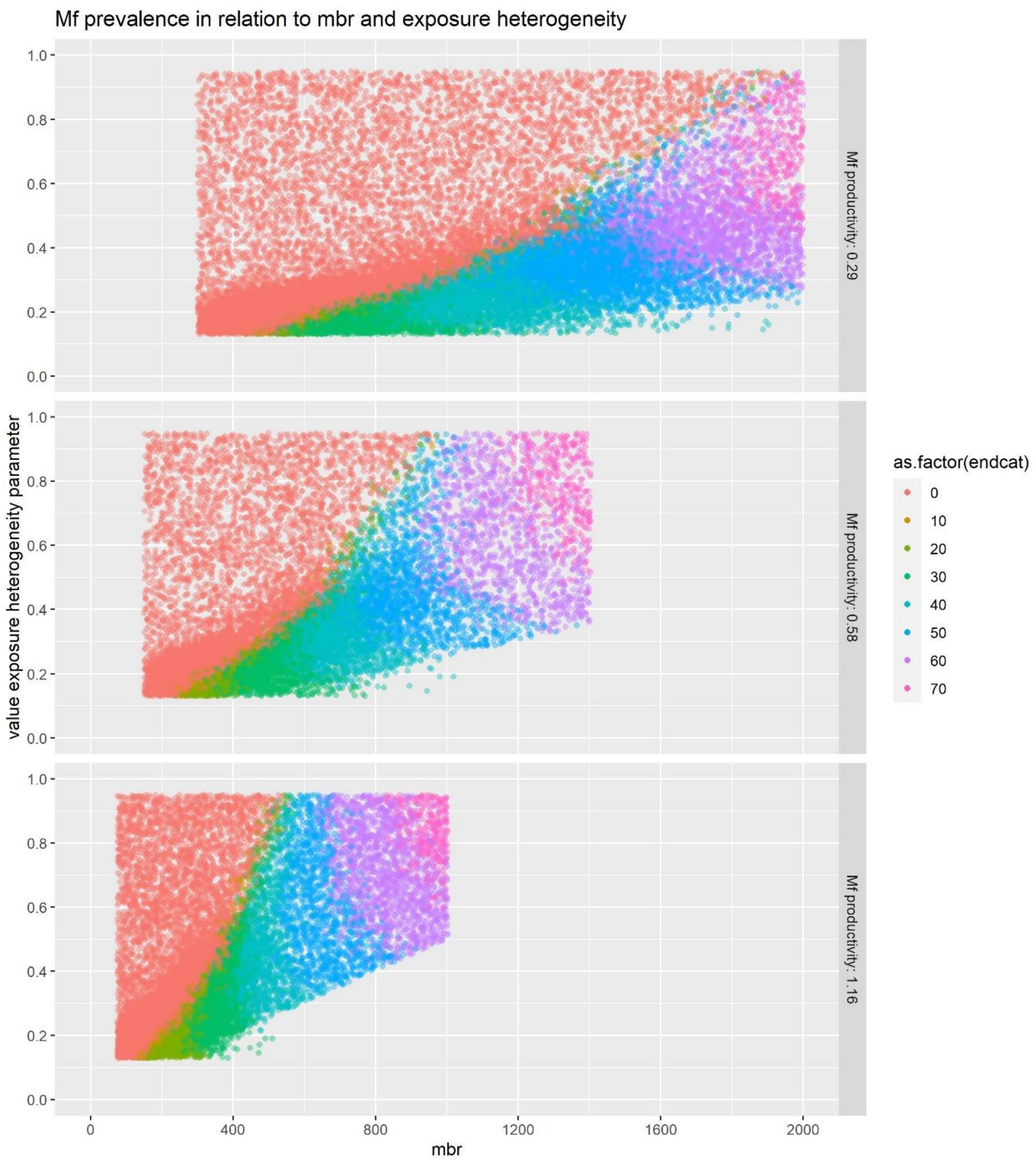

Figure B. Density plot illustrating the parameter space as used in the sensitivity analysis, showing simulated combinations of parameters for the monthly biting rate, the shape (=rate) of the gamma distribution describing exposure heterogeneity in the population (k), and the resulting baseline mf prevalences in 2013 for Côte d'Ivoire. The external force of infection was assumed to be 0 (i.e. no importation from surrounding areas)The model accounts for low-coverage bednet use in the study area since 2006.

Section 2. Validation of model-predicted CFA prevalence levels

2.1 Data

In Côte d'Ivoire, selected communities in the Abengourou health district were treated annually in 2014, 2015, and 2016 by the National Program for the Elimination of Neglected Tropical Diseases, whereas selected villages in Akoupé received biannual treatment [10]. On average, between 60% and 80% of the population reported to be using insecticide-treated nets during the trial (varying between years). Before the onset of the trial, through 2013, the bed net coverage was likely considerably lower [11]. Some of our study communities may have been included in ivermectin MDA for onchocerciasis control, which was provided in the area since 1992 in communities with a population of ≤2000 and within 5 km from a river, with an interruption from 2003-2007 due to civil war [10,12]. The last ivermectin treatment happened at least 12 months before the current study. MDA with ivermectin plus albendazole had not been provided before the onset of the trial.

The Liberia study [13] took place in the Harper district in Maryland County. Selected villages inland were treated annually by teams from the National Public Health Institute of Liberia and the NTD team of the Ministry of Health in 2013, 2014, and 2015, usually in August. Selected villages in the coastal area were treated biannually. The average reported use of insecticide treated bed nets was <25% during the first years of the trial, and increased in the latter years. External sources confirm that bed net usage in the study area was low through 2014 and increased thereafter [11]. The area did not have any previous MDA for LF or onchocerciasis, although a small proportion of the population reported to have been treated with ivermectin at some point before the study.

In both sites, CFA and Mf positivity was assessed by study teams in the consenting population aged 5 years and above, before the first treatment and 11 months after each treatment (i.e. preceding the next treatment). Data from different survey moments could not be matched at the individual level (no individual-level follow-up). In Côte d'Ivoire, CFA was always assessed using FTS, but 2 communities also used ICT at baseline. In Liberia, the ICT test was used in the baseline survey, whereas FTS was used in subsequent surveys (in some communities in combination with ICT). In both study areas, presence of Mf was assessed by microscopic examination of 60 µL finger prick blood obtained at night. Presence of Mf was usually only assessed in CFA-positive individuals, in which case the overall Mf prevalence in the population was estimated assuming that all CFA-negative individuals are also Mf-negative and that the proportion of Mf positives among tested CFA-positives is representative for the total group of CFA positives. Only in Côte d'Ivoire, the full 5+ population was tested during the baseline survey and, in some communities, the first follow-up survey. Community-specific data for a given timepoint and location were included in our analysis if ≥30 individuals were examined by CFA.

2.2 Methods

For model validation, we use baseline data from both the annual and biannual treatment arms. Trends over time are only simulated and compared to data for annual MDA, as biannual MDA is not recommended for LF elimination programmes.

We first assessed whether the model-predicted Mf-CFA prevalence association at baselines matched to observed data (taken from both the annual and biannual treatment arm). To obtain simulation runs across the spectrum of observed baseline endemicity levels, we performed a large number of runs for each scenario, varying the value of the three model parameters relating to setting-specific transmission conditions: the monthly biting rate (i.e. mean number of mosquito bites per adult per month), the degree of interindividual variation in exposure to mosquito bites, and an external force of infection (included to mimic infections acquired from outside the simulated population, either through human mobility or vector mobility). The external force-of-infection was set to zero in half of the simulation runs, to mimic communities where transmission is independent of imported infections. In the other runs, it was set to a low value, varying between runs but constant over time to mimic communities where low endemicity is stabilized by incoming infection from surrounding areas. Values used for these parameters are shown in Figure A (this supplement). We assumed a population size of about 1000 individuals per village.

For the comparison of model-predicted trends to observed data from Côte d'Ivoire and Liberia, we simulated the annual MDA as in the trials as well as local use of bed nets since 2006. Treatment coverage achieved in the trials was not known. We assumed that on average 65% of the total population was treated per round, with treatment only provided to individuals aged 5 years and above. Details of the simulated scenarios are provided in Table B below. For Côte d'Ivoire, we did not account for previous MDA of ivermectin only, of which the last round took place more than 12 months before the baseline survey.

For Figure 2 (main text) and Figure C (this supplement), we selected per community the subset of runs with Mf and CFA prevalence at baseline falling within an ellipse around the observed value, defined by the 95% confidence interval around the observed Mf and CFA prevalence, and model-predicted Mf and CFA prevalences for later timepoints were then compared to data.

Table B. Simulated scenarios for comparing model predictions to data from Côte d'Ivoire and Liberia

Calendar year	Côte d'Ivoire		Liberia	
	Assumed bednet coverage[a](apply to whole year)	MDA coverage[b,c] (% out of total population)	Assumed bednet coverage[a](apply to whole year)	MDA coverage[b] (% out of total population)
2006	0%	-	20%	-
2007	7%	-	23%	-
2008	11%	-	24%	-
2009	10%	-	24%	-
2010	10%	-	35%	-
2011	39%	-	25%	-
2012	17%	-	27%	-
2013	12%	-	19%	65%
2014	63%	65%	20%	65%
2015	90%	65%	60%	65%
2016	79%	65%	60%	0%
2017	62%	0%	60%	0%
2018	62%	-	60%	-

[a] We assumed that this bednet coverage applied to the whole year.
[b] Number of people treated (all aged 5 years and above) out of the total population (including children under 5 years of age). We assume 5% systematic non compliance, meaning that 5% of the total population never participates in MDA.
[c] The simulations do not account for the impact of annual ivermectin MDA in Côte d'Ivoire that was provided for the control of onchocerciasis in the area since 1992, with an interruption from 2003-2007 due to civil war [10,12]

Section 3. PRIME-NTD table: Policy-Relevant Items for Reporting Models in Epidemiology of Neglected Tropical Diseases

Table C. The Policy-Relevant Items for Reporting Models in Epidemiology of Neglected Tropical Diseases (PRIME-NTD) [a]

Principle [b]	What has been done to satisfy the principle?	Where in the manuscript is this described?
1. Stakeholder engagement	Key stake holders in this work include global-level policy makers, and people involved in monitoring & evaluation of LF elimination programmes. Representatives of the latter groups participated in the work and are co-author of the paper.	Author list, author contribution, acknowledgements section
2. Complete model documentation	We used a previously published model, that has been described elsewhere in detail. We included a brief model description in the current paper, with references to original model description papers. A link to the publicly available source code is given in the section 1 in this supplement. We provided full information on the quantification of all model parameters pertaining to transmission, life history and productivity of the parasite, morbidity, vector, drug efficacy, treatment histories (timing, frequency and coverage of mass treatment), and surveys.	Methods section; section 1 in this supplement
3. Complete description of data used	This paper used empirical data to verify that model-predicted patterns in mf and CFA prevalence match to empirically observed patterns. A summary description of the study area and data is included in the methods section with references to earlier publications if available. Scenarios derived from these data are provided in section 2 in this supplement. Relevant summary data, to which model predictions are compared, are included in figures in the manuscript and supplement.	Methods section; section 2 in this supplement; figures 1 and 2 main text; figures C, D, E and L in this supplement
4. Communicating uncertainty	We included figures showing stochastic variation in model-predicted trends in mf prevalence. We also showed how the predictive value of possible thresholds depend on baseline endemicity and MDA history. We assessed the sensitivity of model outcomes to changes in the assumed mf productivity rate per female worm and the number of people sampled.	Results; figures F-M in this supplement
5. Testable model outcomes	We tested whether the model-predicted CFA-mf prevalence association is in line with data, both at baseline and at different follow-up moments. Model predictions pertaining to the situation after the study period could be tested against data, should relevant data be collected. Predictions about the achievement of true elimination 50 years after the last treatment round are difficult to test against real data in practice.	Figure 1 main text; figures C, D, E and L in this supplement

[a] Communication of adherence to the five principles of the NTD Modelling Consortium for policy-relevant work, described in: Behrend et al. 2020. Modelling for policy: The five principles of the Neglected Tropical Diseases Modelling Consortium. *PLoS Negl Trop Dis* 2020; **14**(4): e0008033.

[b] Full formulation of the principles:

1. Don't do it alone. Engage stakeholders throughout, from the formulation of questions to the discussions on the implications of the findings.
2. Reproducibility is key! Prepare and make available (preferably open-source) a complete technical documentation of all model code, mathematical formulas, assumptions and their justification, allowing others to reproduce the model.
3. Model calibration, goodness-of-fit and validation are fundamental processes of scientific modelling. All data used should be described in sufficient detail to allow the reader to assess the type and quality of these analyses. When using data by reference, use Principle 2.
4. Communicating uncertainty is a hallmark of good modelling practice. Perform a sensitivity analysis of all key parameters, and for each paper reporting model predictions include an uncertainty assessment of those model outputs within the paper.
5. Model outcomes should be articulated in the form of testable hypotheses. This allows comparison with other models and future events as part of the ongoing cycle of model improvement.

Section 4. Detailed results

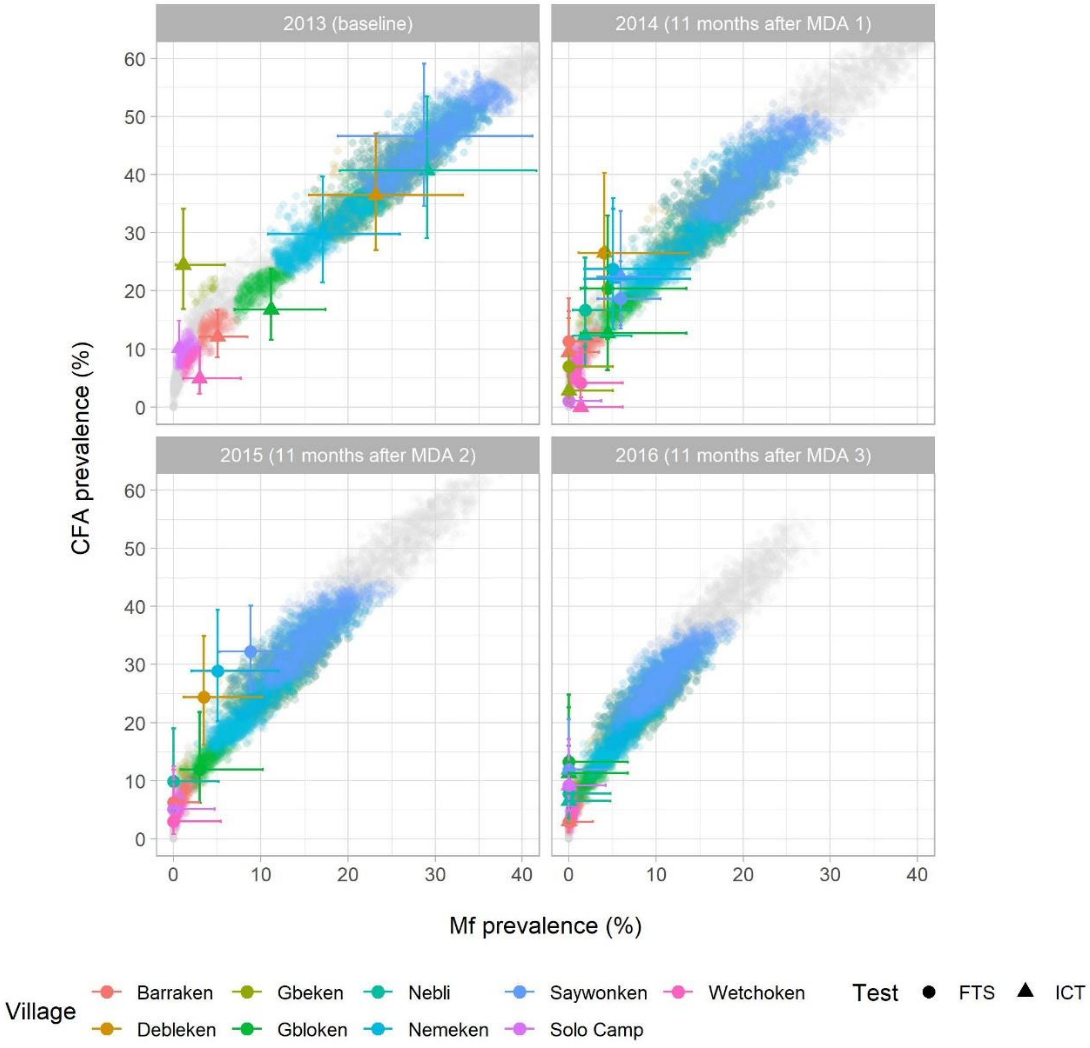

Figure C. Observed and simulated prevalence of microfilaraemia (Mf) and circulating filarial antigenaemia (CFA) for Liberia, at baseline (2013, before the first treatment round) and in 2014, 2015 and 2016 (i.e. 11 months after the first, second and third MDA round). FTS- and ICT-based observations are shown as circles and triangles, respectively, along with 95% confidence intervals. Note that both test were used simultaneously in some villages at follow-up moments 1 and 3, and confidence intervals are presented as two-barred crosses. Model predictions are shown as small dots. Simulation results from runs matched to specific villages at baseline are shown in the color of that village, and remaining runs are shown in lightgrey. A run was considered a match if the predicted Mf-CFA prevalence combination at baseline fell within the ellipse drawn around the observed MF-CFA prevalence combination based on the 95% confidence intervals. For both the models and the observed data, crude prevalence estimates are presented in the figures (i.e. not age-standardized). The MDA coverage was assumed to be 65% of the total population per round in the simulation runs. See Table B (this supplement) for details about the simulated scenarios, and see Figure 2 in the main text for a similar figure for Côte d'Ivoire.

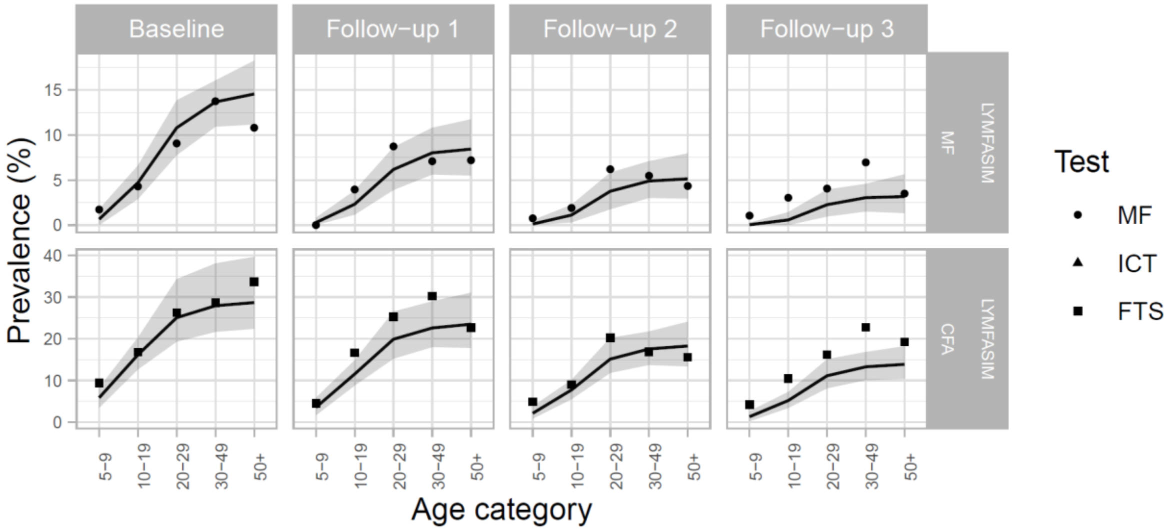

Figure D. Observed and simulated prevalence of microfilaraemia (Mf) and circulating filarial antigenaemia (CFA, measured by filarial test strip) by age for Côte d'Ivoire, at baseline (before the first treatment round) and at follow-up moments 1, 2 and 3 (11 months after MDA rounds 1, 2, and 3, respectively). Model predictions (grey shaded area) are shown for the subset of simulations, of which the overall Mf prevalence fell within the 95% confidence interval around the observed mean prevalence, without selecting the corresponding CFA prevalence. The MDA coverage was assumed to be 65% of the total population. See Table B (this supplement) for details about the simulated scenarios.

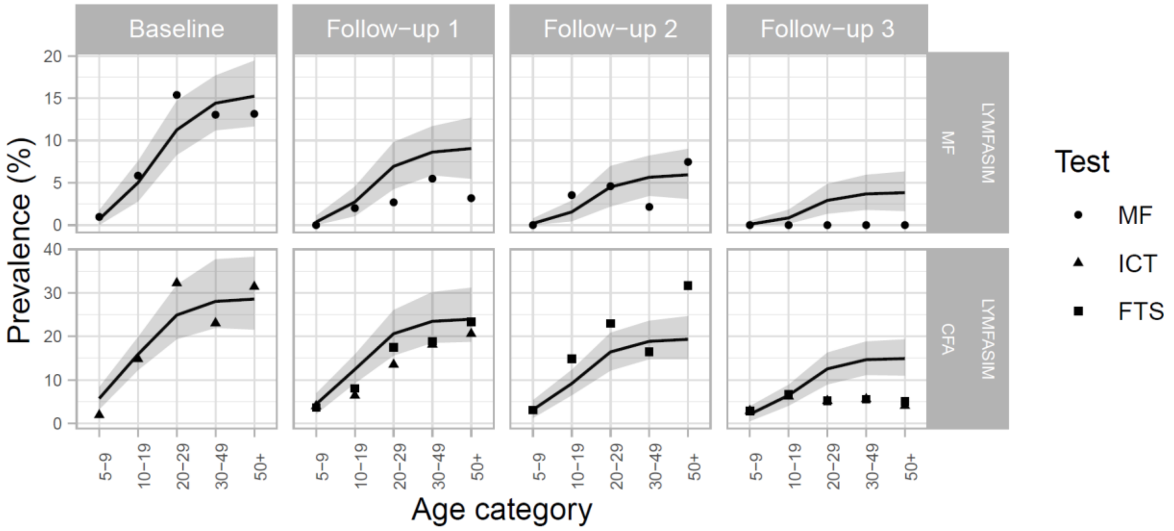

Figure E. Observed and simulated prevalence of microfilaraemia (Mf) and circulating filarial antigenaemia (CFA) by age for Liberia, at baseline (before the first treatment round) and at follow-up moments 1, 2 and 3 (11 months after MDA rounds 1, 2, and 3, respectively). FTS- and ICT-based observations are shown as black squares and triangles, respectively. Note that both CFA test were used at follow-up moments 1 and 3. Model predictions (grey shaded area) are shown for the subset of simulations, of which the overall Mf prevalence fell within the 95% confidence interval around the observed mean prevalence, without selecting the corresponding CFA prevalence. The MDA coverage was assumed to be 65% of the total population. See Table B in this supplement for details about the simulated scenarios.

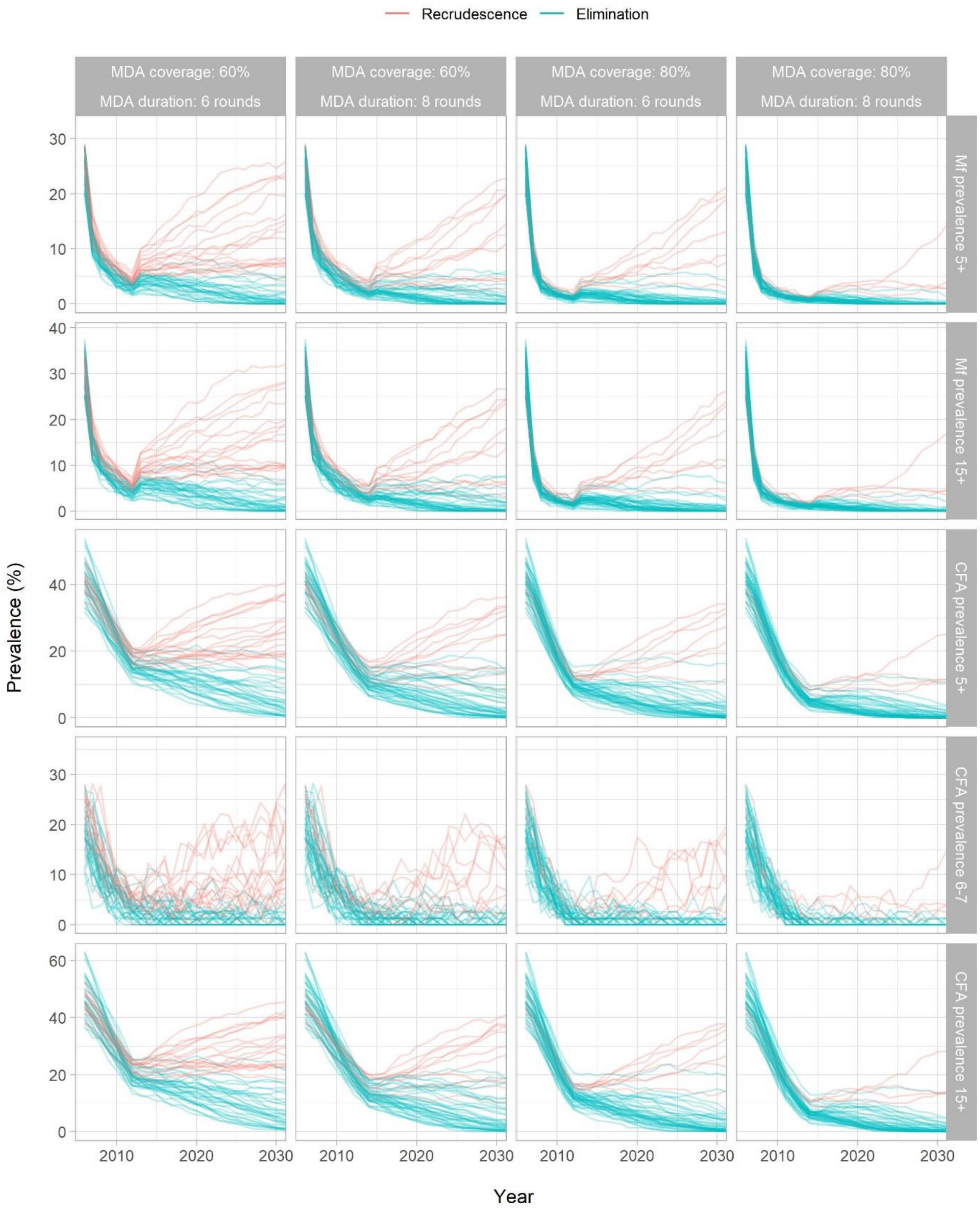

Figure F. Trends in infection indicators as predicted by LYMFASIM during and after mass drug administration (MDA), for communities with **microfilaraemia (Mf) prevalence at baseline varying between 20%-30%**. Results are presented for four different MDA scenarios (in columns: 6 or 8 rounds of MDA with ivermectin+albendazole, with 60% of 80% coverage), five different indicators of infection (rows: Mf and CFA prevalence in the 5+ and 15+ population, CFA prevalence in 6-7-year olds). Each line presents the outcome of a single simulation run, with runs eventually ending in elimination shown in blue and runs ending in resurgence shown in red. Results are shown for 50 runs, randomly selected from the runs that were done to calculate receiver operating characteristic (ROC) curves and positive and negative predictive value of each of the three indicator for predicting elimination.

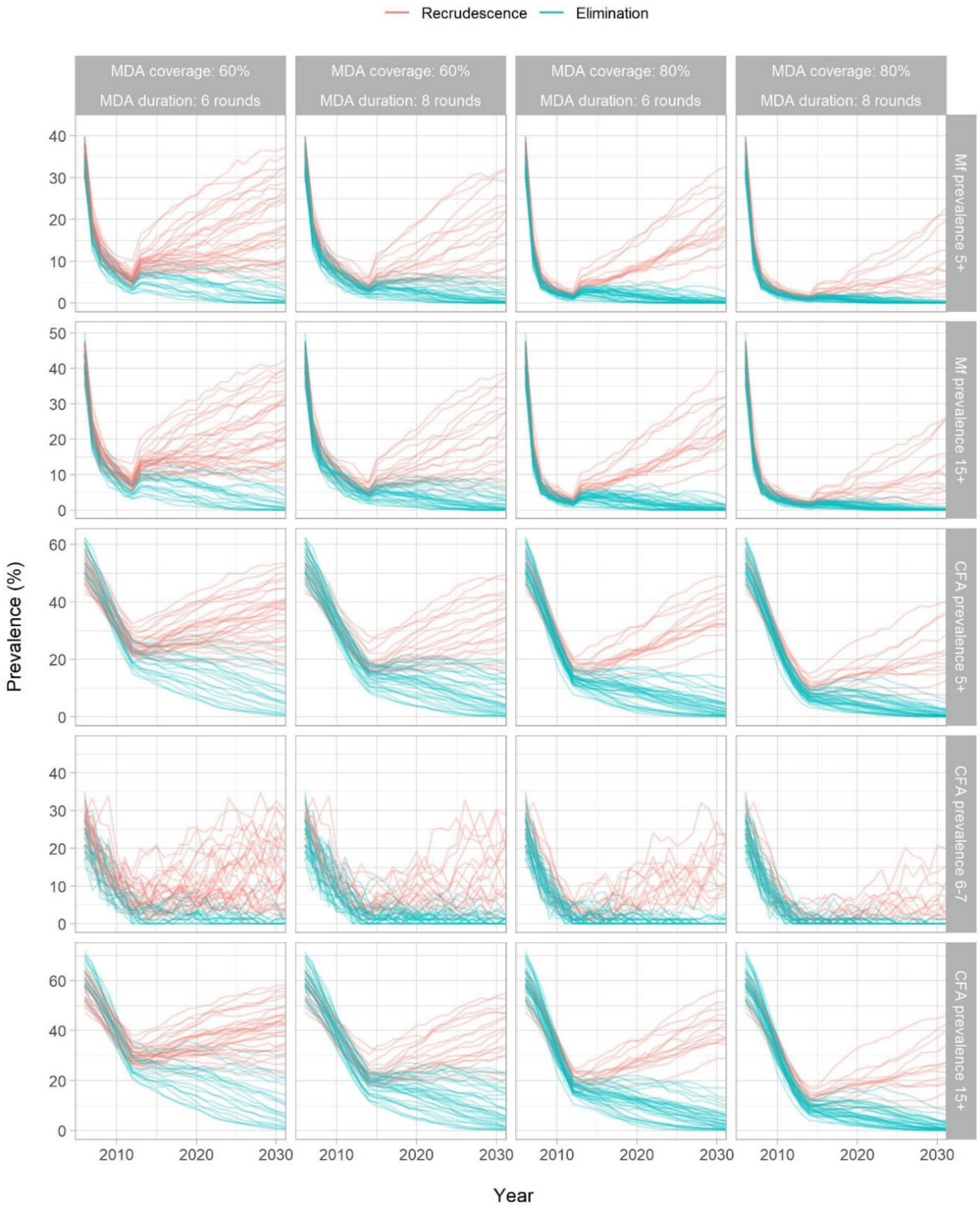

Figure G. Trends in infection indicators as predicted by LYMFASIM during and after mass drug administration (MDA), for communities with **microfilaraemia (Mf) prevalence at baseline varying between 30%-40%**. Results are presented for four different MDA scenarios (in columns: 6 or 8 rounds of MDA with ivermectin+albendazole, with 60% of 80% coverage), five different indicators of infection (rows: Mf and CFA prevalence in the 5+ and 15+ population, CFA prevalence in 6-7-year olds). Each line presents the outcome of a single simulation run, with runs eventually ending in elimination shown in blue and runs ending in resurgence shown in red. Results are shown for 50 runs, randomly selected from the runs that were done to calculate receiver operating characteristic (ROC) curves and positive and negative predictive value of each of the three indicator for predicting elimination.

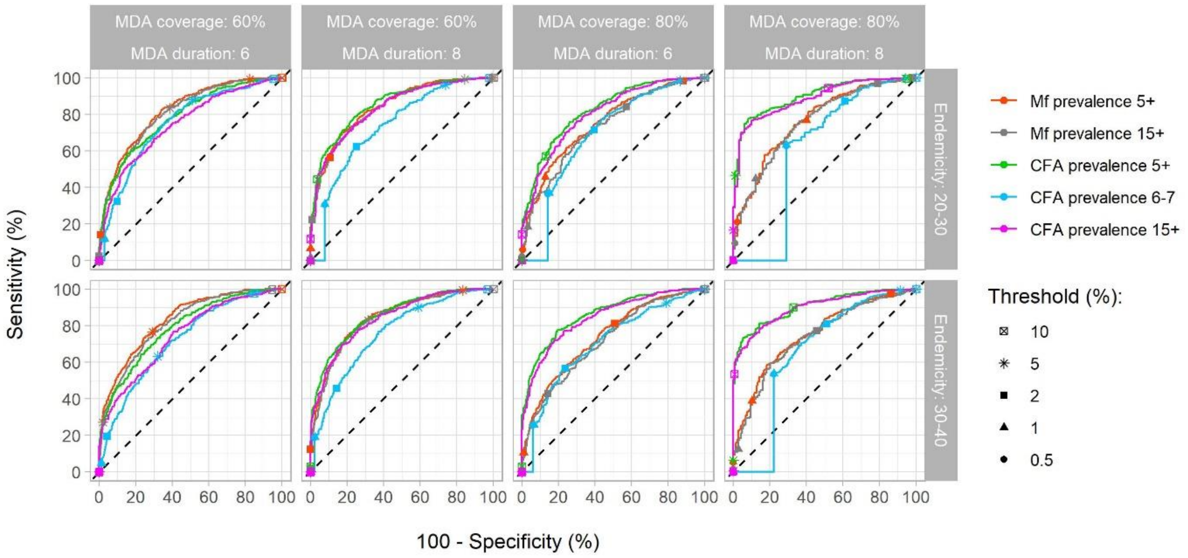

Figure H. Receiver-operator characteristic (ROC) curves for predicting the eventual occurrence of elimination of transmission, based on the microfilaraemia (Mf) or circulating filarial antigenaemia (CFA) prevalence measured 1 year after the last treatment round, for different MDA scenarios. Based on predictions from the LYMFASIM model. Results are shown by treatment scenario and for different endemicity categories, with the latter based on Mf prevalence at baseline. Different lines show the predictive performance of the Mf prevalence among the 5+ population (red line), the CFA prevalence measured in the 5+ population (green line) and the CFA prevalence measured among 6-7-year-old children (blue line). Sensitivity is the percentage of simulation runs ending in elimination that are correctly identified based on Mf or CFA prevalence below a range of thresholds (see legend). Similarly, 100%-specificity is the percentage of simulation runs resulting in resurgence, which is not correctly identified. The optimal situation is in the upper left corner of the panels (100% sensitivity and 100% specificity).

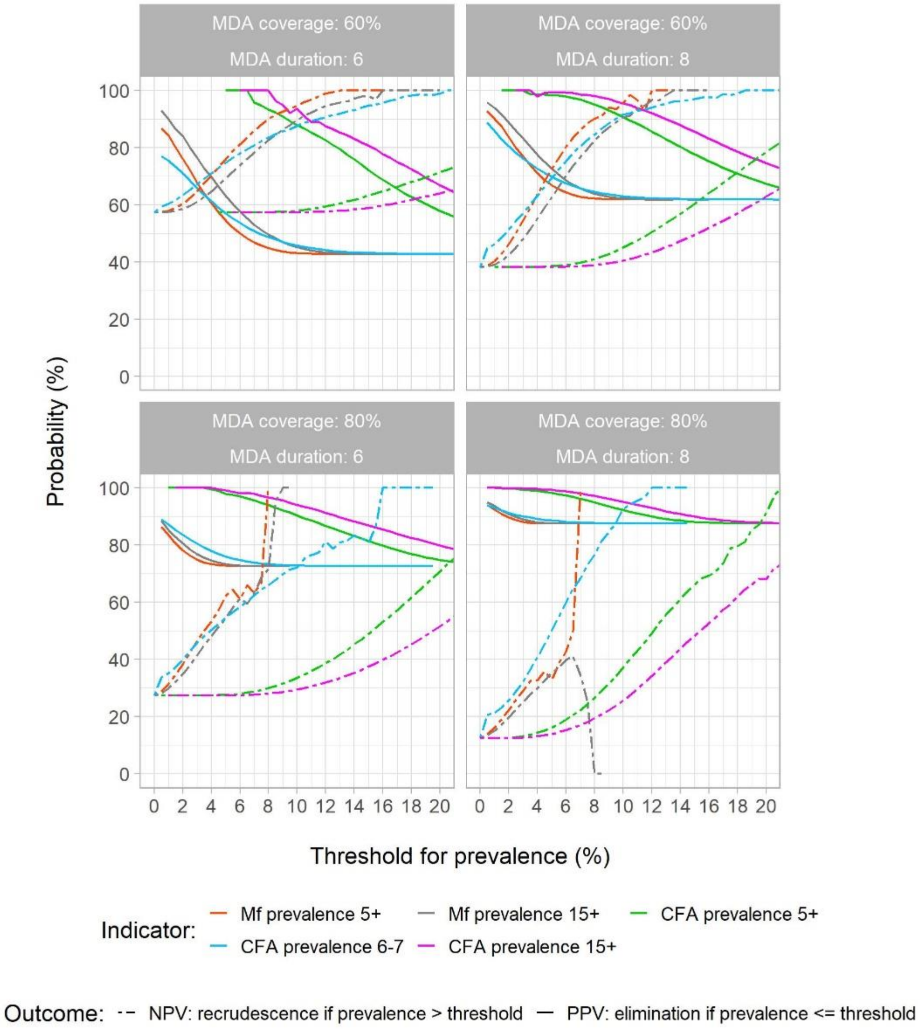

Figure I. Positive predictive value (PPV, probability of achieving elimination within 50 years after the last round of MDA, if the 1-year post MDA prevalence measured in 200 individuals sampled per age group was below the threshold) and negative predicted value (NPV, probability of recrudescence, if the 1-year post MDA prevalence measured in 200 individuals sampled per age group was above the threshold) for a range of possible thresholds. Different lines show the predictive performance of the Mf and CFA prevalence in the 5+ or 15+ population (MF: red and grey line; CFA: green and pink lines) and the CFA prevalence in 6-or-7-year-old children (blue line). Based on 1000 simulations per scenario and endemicity category, with baseline Mf prevalence varying between 20%-30% or between 30%-40%, and assuming that 200 individuals are sampled per age group.

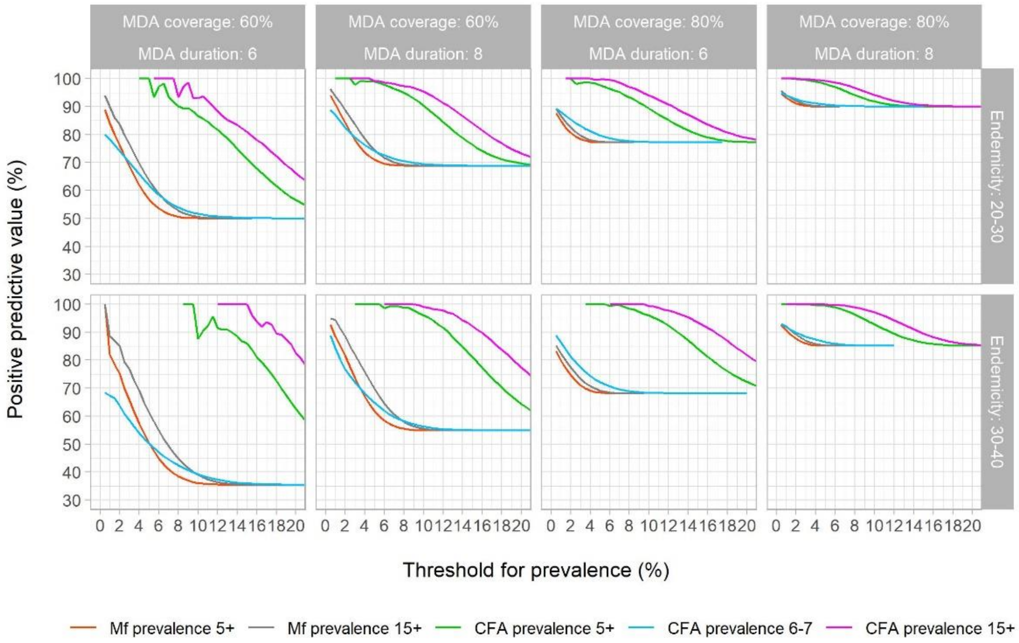

Figure J. Probability of achieving elimination within 50 years after the last round of MDA, if the 1-year post MDA prevalence measured in 200 individuals sampled per age group was below a given threshold (i.e. the positive predictive value (PPV) of using this threshold), in relation to baseline endemicity level. Different lines show the predictive performance of the Mf and CFA prevalence in the 5+ or 15+ population (MF: red and grey line; CFA: green and pink lines) and the CFA prevalence in 6-or-7-year-old children (blue line). Based on 1000 simulations per scenario and endemicity category, with baseline Mf prevalence varying between 20%-30% or between 30%-40%.

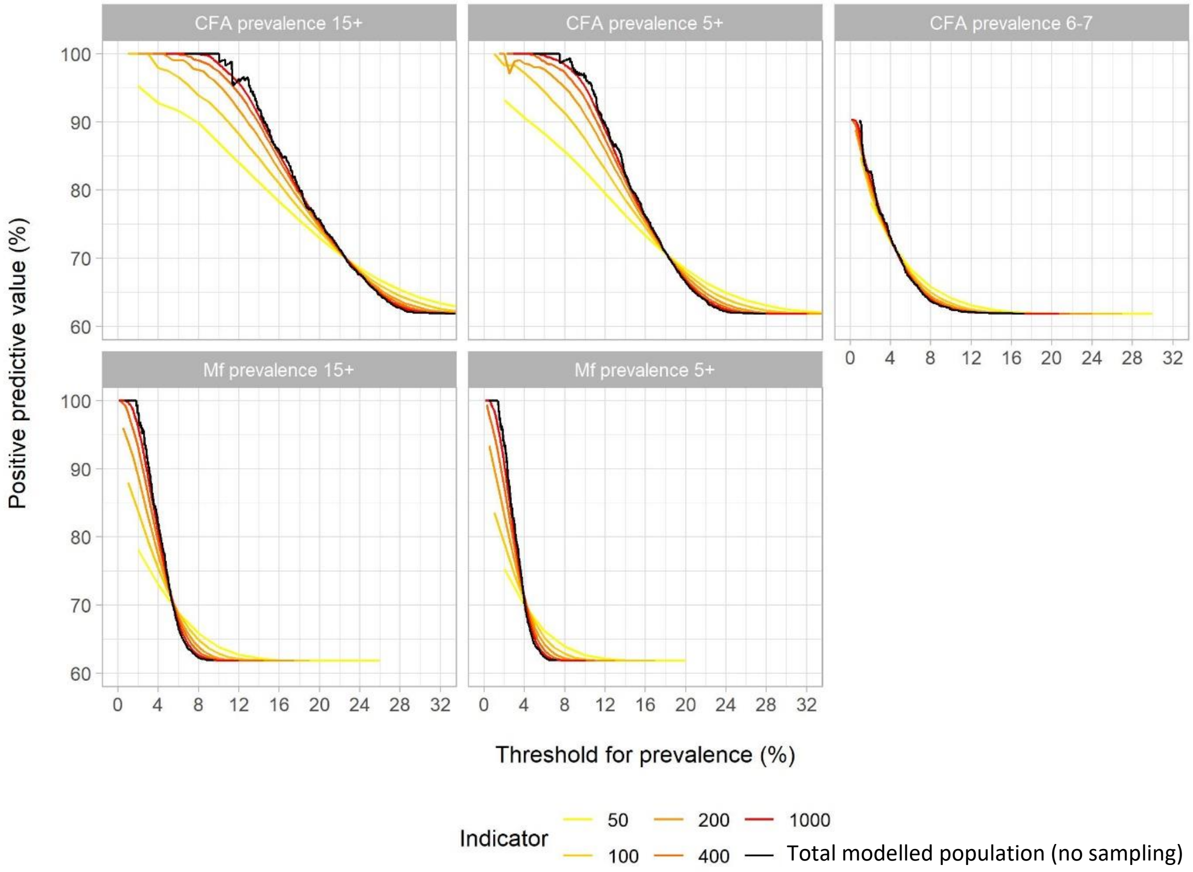

Figure K. Probability of achieving elimination within 50 years after the last round of MDA, if the 1-year post MDA prevalence was below a given threshold (i.e. the positive predictive value (PPV) of using this threshold) in relation to the number of people sampled. Results for different indicators are shown in separate panels (CFA or MF prevalence in people aged ≥15 or ≥5 years, CFA prevalence in 6-or-7-year-old children). Based on 2000 simulations per scenario, with baseline Mf prevalence varying between 20%-40%.

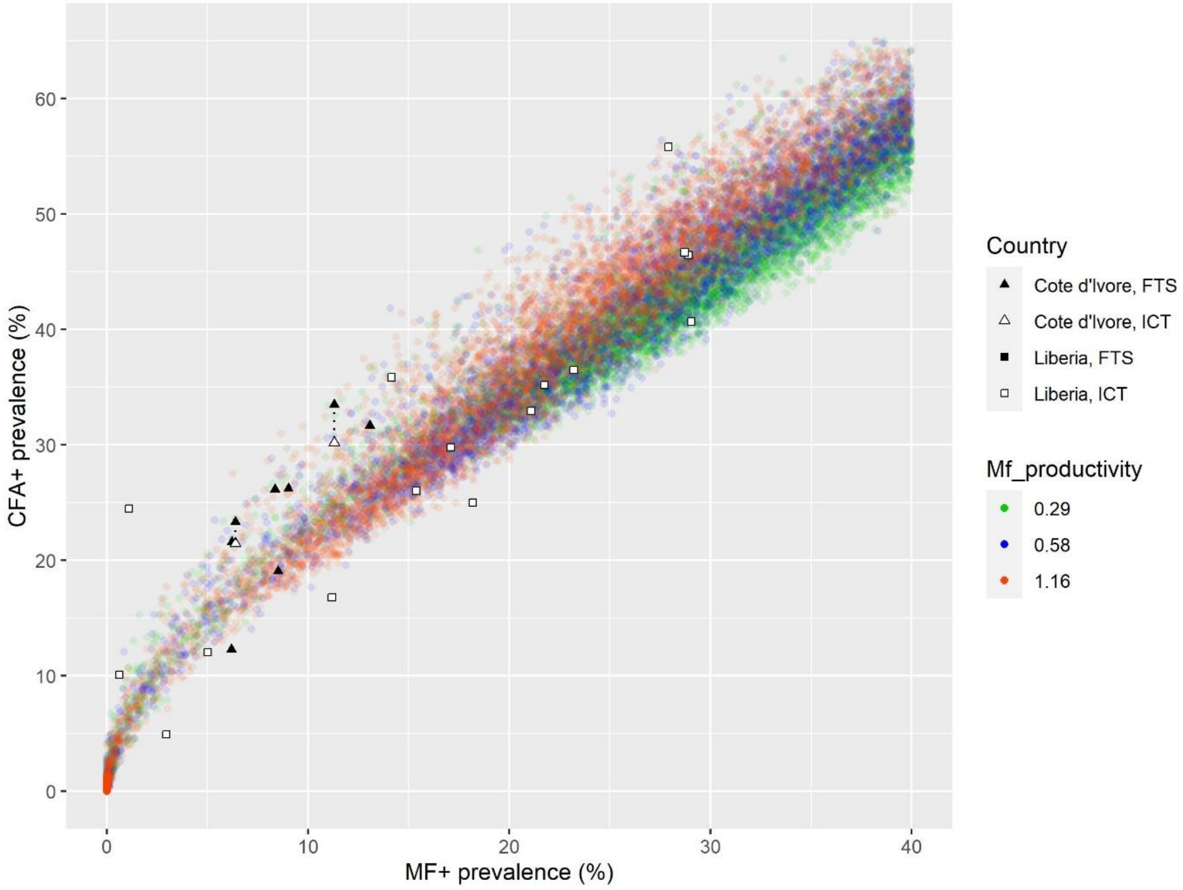

Figure L. Comparison of the model-predicted and observed association between microfilaraemia (Mf) and circulating filarial antigenaemia (CFA) prevalence at baseline, varying model assumptions regarding the monthly rate of Mf production per female worm per 20µL blood. Observed data from Côte d'Ivoire (triangles) and Liberia (squares) were based on surveys performed before the introduction of MDA, including data from both the annual and biannual treatment arm with Mf and CFA prevalence measured about one month before the first round of MDA in the trial (i.e. 2014 for Côte d'Ivoire and 2013 for Liberia). Model predictions are shown for settings without (dark blue) and with (light blue) an external force of infection, accounting for low-coverage bednet use since 2006 based on data from Côte d'Ivoire.

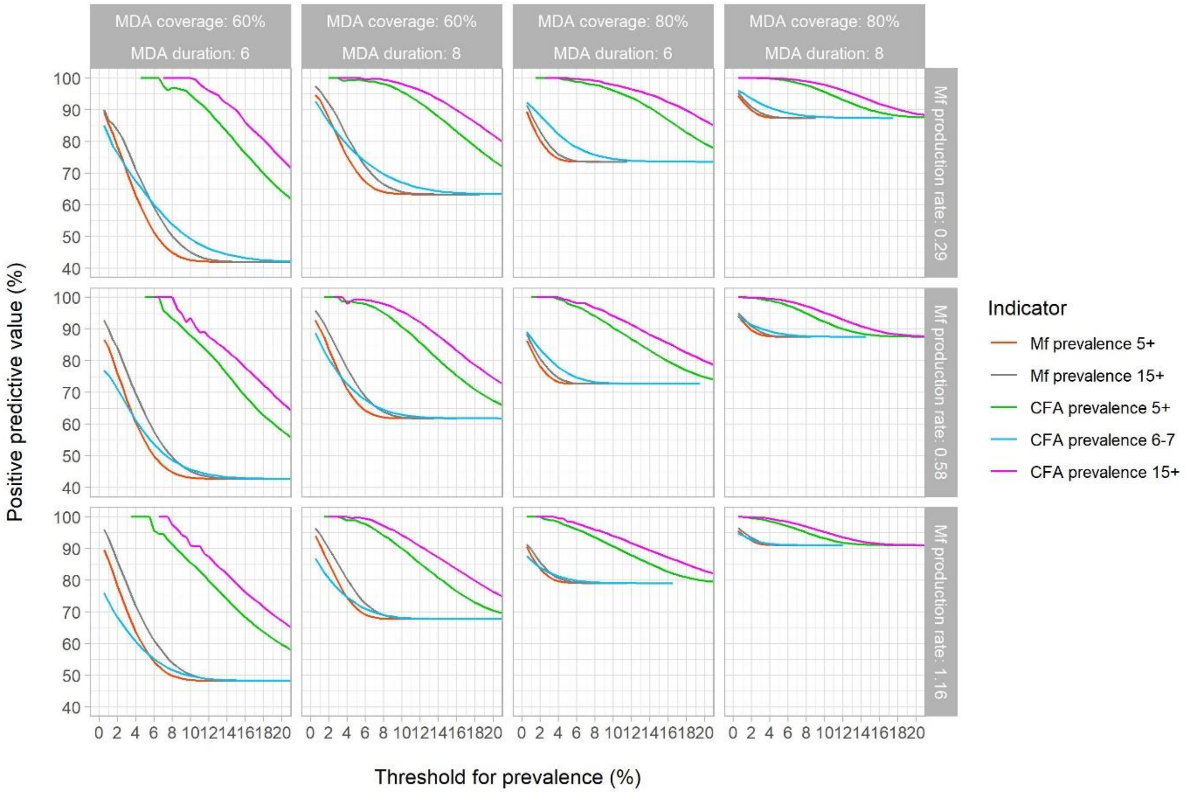

Figure M. Model-predicted probability of achieving elimination within 50 years after the last round of MDA, if the 1-year post MDA prevalence was below a given threshold (i.e. the positive predictive value of using this threshold), under different assumptions regarding the monthly rate of Mf production per female worm per 20µL blood. Different lines show the predictive performance of the Mf and CFA prevalence in a sample of 200 individuals taken from the 5+ or 15+ population (MF: red and grey line; CFA: green and pink lines) and the CFA prevalence in a sample of 200 children 6 or 7 years or (blue line). Based on 2000 simulations per scenario, with baseline Mf prevalence varying between 20% and 40%.

References

1. Plaisier AP, Subramanian S, Das PK, Souza W, Lapa T, et al. (1998) The LYMFASIM simulation program for modeling lymphatic filariasis and its control. Methods of Information in Medicine 37: 97-108.

2. Coffeng LE, Bakker R, Montresor A, de Vlas SJ (2015) Feasibility of controlling hookworm infection through preventive chemotherapy: a simulation study using the individual-based WORMSIM modelling framework. Parasit Vectors 8: 541.

3. Subramanian S, Stolk WA, Ramaiah KD, Plaisier AP, Krishnamoorthy K, et al. (2004) The dynamics of *Wuchereria bancrofti* infection: a model-based analysis of longitudinal data from Pondicherry, India. Parasitology 128: 467-482.

4. Stolk WA, ten Bosch QA, de Vlas SJ, Fischer PU, Weil GJ, et al. (2013) Modeling the impact and costs of semiannual mass drug administration for accelerated elimination of lymphatic filariasis. PLoS Negl Trop Dis 7: e1984.

5. Smith ME, Singh BK, Irvine MA, Stolk WA, Subramanian S, et al. (2017) Predicting lymphatic filariasis transmission and elimination dynamics using a multi-model ensemble framework. Epidemics 18: 16-28.

6. Stolk WA, Prada JM, Smith ME, Kontoroupis P, de Vos AS, et al. (2018) Are Alternative Strategies Required to Accelerate the Global Elimination of Lymphatic Filariasis? Insights From Mathematical Models. Clin Infect Dis 66: S260-S266.

7. Stolk WA, de Vlas SJ, Borsboom GJ, Habbema JD (2008) LYMFASIM, a simulation model for predicting the impact of lymphatic filariasis control: quantification for African villages. Parasitology 135: 1583-1598.

8. World Health Organization (1992) Lymphatic filariasis: the disease and its control. Fifth report of the WHO Expert Committee on Filariasis. World Health Organ Tech Rep Ser 821: 1-71.

9. Plaisier AP, Cao WC, van Oortmarssen GJ, Habbema JD (1999) Efficacy of ivermectin in the treatment of *Wuchereria bancrofti* infection: a model-based analysis of trial results. Parasitology 119: 385-394.

10. Loukouri A, Meite A, Koudou BG, Goss CW, Lew D, et al. (2020) Impact of annual and semi-annual mass drug administration for Lymphatic Filariasis and Onchocerciasis on Hookworm Infection in Cote d'Ivoire. PLoS Negl Trop Dis 14: e0008642.

11. Author Malaria Atlas Project: interactive map of insecticide-treated bednet (ITN) coverage in Africa, 2000-2015. Malaria Atlas Project. Available: https://malariaatlas.org/ [Accessed 9 December 2020 Cited].

12. Koudou BG, Kouakou MM, Ouattara AF, Yeo S, Brika P, et al. (2018) Update on the current status of onchocerciasis in Cote d'Ivoire following 40 years of intervention: Progress and challenges. PLoS Negl Trop Dis 12: e0006897.

13. Eneanya OA, Gankpala L, Goss CW, Momolu AT, Nyan ES, et al. (2022) Community-based trial assessing the impact of annual versus semiannual mass drug administration with ivermectin plus albendazole and praziquantel on helminth infections in northwestern Liberia. Acta Trop 231: 106437.
